# Diversity, Chemical Constituents, and Biological Activities of Endophytic Fungi Isolated From *Ligusticum chuanxiong* Hort

**DOI:** 10.3389/fmicb.2021.771000

**Published:** 2021-11-17

**Authors:** Zizhong Tang, Yihan Qin, Wenhui Chen, Zhiqiao Zhao, Wenjie Lin, Yirong Xiao, Hong Chen, Yuntao Liu, Hui Chen, Tongliang Bu, Qingfeng Li, Yi Cai, Huipeng Yao, Yujun Wan

**Affiliations:** ^1^College of Life Sciences, Sichuan Agricultural University, Ya’an, China; ^2^Sichuan Agricultural University Hospital, Ya’an, China; ^3^College of Food Science, Sichuan Agricultural University, Ya’an, China; ^4^Sichuan Food Fermentation Industry Research and Design Institute, Chengdu, China

**Keywords:** chuanxiong, phenolic compounds, endophytic fungi, antioxidant activity, antimicrobial activity

## Abstract

The objective of this study was to evaluate the diversity of endophytic fungi of different parts of *Ligusticum chuanxiong* Hort (CX) and further characterize their biological activities and identify chemical compounds produced by these endophytic fungi. A total of 21 endophytic fungi were isolated and identified from CX. *Penicillium oxalicum*, *Simplicillium* sp., and *Colletotrichum* sp. were identified as promising strains by the color reaction. Comparing different organic extracts of the three strains, it was observed that the ethyl acetate extract of *Penicillium oxalicum* and *Simplicillium* sp. and the *n*-butanol extract of *Colletotrichum* sp. showed significant antioxidant and antibacterial activities. The ethyl acetate extracts of *Penicillium oxalicum* had outstanding antioxidant and antibacterial effects, and its radical scavenging rates for ABTS and DPPH were 98.43 ± 0.006% and 90.11 ± 0.032%, respectively. At the same time, their IC_50_ values were only 0.18 ± 0.02 mg/mL and 0.04 ± 0.003 mg/mL. The ethyl acetate extract of *Penicillium oxalicum* showed MIC value of only 0.5 mg/mL against *Escherichia coli* and *Staphylococcus aureus*. By liquid chromatography-mass spectrometry (LC-MS), we found that *Penicillium oxalicum* could produce many high-value polyphenols, such as hesperidin (36.06 μmol/g), ferulic acid (1.17 μmol/g), and alternariol (12.64 μmol/g), which can be a potential resource for the pharmaceutical industry. In conclusion, these results increase the diversity of CX endophytic fungi and the antioxidant and antibacterial activities of their secondary metabolites.

## Introduction

The medicinal plant *Ligusticum chuanxiong* Hort (CX) belongs to the Umbelliferae family and is commonly cultivated in Sichuan Province, China. It has been used in the treatment of gynecological diseases, headache, rheumatism arthralgia, and hemiplegia (Chen et al., 2018). The major chemical constituents of CX are phthalides, terpenes, polysaccharides, alkaloids, and organic acids (Ran et al., 2011). Moreover, the young leaf and stem of CX are widely used as edible food materials in tossed salad and fried cuisines (Yuan et al., 2020a).

Endophytic fungi are a valuable resource for the detection of new natural products and have been widely used for medicinal purposes (Tanvir et al., 2017). Endophytic fungi that live with plant tissues without causing harm to hosts can produce different secondary metabolites including alkaloids, steroids, terpenoids, flavonoids, aliphatic compounds, and phenols (Aly et al., 2011). These products have various biological activities, such as antioxidant, anti-diabetic (Sharma and Sharma, 2016), anti-obesity (Noinart et al., 2017), and antimicrobial activities (Padhi et al., 2020). Moreover, plant endophytic fungi have a high species diversity, which is strongly associated with environmental variations and the taxonomy of host plants (Wu et al., 2013). The diversity of endophytic fungi will determine the production of diverse secondary metabolites promoted by endophytic fungi, which can be used as drugs for medical treatment (Jia et al., 2016). Furthermore, when the endophytic fungi was isolated *in vitro* and cultivated in suitable conditions, the number of fungal cells and the production of secondary metabolites would be multiplied far beyond their normal concentration in plants (Wu et al., 2018). Therefore, plant endophytic fungi are not only a source for the discovery of biologically active substances, but also a reactor for the production of drugs more easily and economically.

Currently, oxidative stress is gaining more attention. Reactive oxygen species (ROS) are continuously produced as a by-product of normal basal metabolism in humans (Valko et al., 2006). ROS is essential and beneficial to living systems, and plays a role as signaling molecules in the regulation of many biological processes (Apel and Hirt, 2004). However, oxidative stress is the result of excess unneutralized active substances in the body, which can damage cellular lipids, proteins, cells, tissues, and organs, and inhibit the normal function of DNA (Valko et al., 2007; Desai et al., 2009), and it has been reported as one of the important factors in several types of diseases, such as diabetes and even some types of cancer (Marseglia et al., 2015; Zimta et al., 2019). Although many potent antioxidants have been artificially synthesized, such as BHA and BHT, they are not safe as some natural antioxidants (Kahl and Kappus, 1993). Thus, there is a need to find more natural antioxidants to meet the demands of the food and pharmaceutical industries.

In addition, the quest for new antimicrobial drugs has always been crucial throughout human history. Bacterial infections are the result of pathogenic bacteria entering and multiplying in the human body; these are a major deleterious factor in some infections (Anderson and Kendall, 2017; Martin and Bachman, 2018). For example, the gram-positive bacterium *Staphylococcus aureus* is a human pathogen (Kimmig et al., 2021) that is constantly a source of skin infections, lung infections, gastroenteritis, and urinary tract infections (Thomer et al., 2016; Del Giudice, 2020). Over the years, researchers have observed that antibacterial substances can be isolated from endophytic fungi of various medicinal plants (Pinheiro et al., 2017; Nischitha and Shivanna, 2021). Hence, it is possible to discover new and effective antibacterial substances from endophytic fungi.

Medicinal plants and their endophytes are important resources for the discovery and acquisition of natural products (Huang et al., 2007). The antioxidant (Ge et al., 2018) and antimicrobial (Sim and Shin, 2008) activities of CX have been described using different pharmacological models, and CX is also well documented for production of various bioactive metabolites (Yuan et al., 2020b; Zhang et al., 2020). However, there are few studies on endophytic fungi of CX at present, and endophytic fungi resources should be further developed and utilized. In this context, the present study aimed to identify the diversity of endophytic fungi in CX and the antioxidant and antibacterial activities as well as the chemical compounds of endophytic fungus extracts.

## Materials and Methods

### Experimental Materials

Seedlings of CX were collected from the farm of Sichuan Agricultural University (Ya’an City, Sichuan Province, China) in October 2020 and the lingzi (nodes of the lower part of the stems) were obtained from the local market in Ya’an. The plants were identified by Dr. Hui Chen. Voucher specimens were deposited in the Herbarium “Fermentation Engineering Laboratory” with the identification numbers FEL125 and EFL126. All samples were placed in the boxes and immediately sent to the laboratory for study.

### Isolation of Endophytic Fungi

The lingzi and seedlings of CX were washed thoroughly with water, and the roots, stems, and leaves systems were separated from the plant. The collected samples were cut into small 5 mm cubes and soaked in 70% ethanol for 1–2 min, followed by 5% sodium hypochlorite for 2–5 min, and then in 70% ethanol for 0.5–1 min (adapted from Zhao et al., 2012). In addition, the samples were dabbed with sterile filter paper. The surface-sterilized explant fragments were placed in Petri dishes containing PDA which was amended with ampicillin and kanamycin sulfate at a concentration of 50 μg/L to prevent bacterial contamination. The fungal strains in PDA plates were incubated at 28°C for 7–10 days and purified promising fungi were stored in the Laboratory of Molecular Biology and Biochemistry, College of Life Sciences, Sichuan Agricultural University.

### Identification and Phylogenetic Analysis of the Endophytic Fungi

The endophytic fungi were identified by their morphology and DNA sequence data. Cellular morphological features including their mycelium color, pigmentation, and spore morphology, were described and investigated using a CX21 FS1 microscope (Olympus, Tokyo, Japan). Genomic DNA was isolated using the CTAB method and the ITS region of the fungal genome was amplified by PCR using the following primers: ITS1-forward primer (TCCGTAGGTGAACCTGCGG) and ITS4-reverse primer (TCCTCCGCTTATTGATATGC). The reaction mixture and amplification conditions were the same as previously described (Shah et al., 2019). The PCR products were directly sequenced by the TSINGKE Biological Technology Corporation (Shandong, China). The sequences of the ITS regions were compared with the sequences of existing species sequence in GenBank using BLASTN software. A phylogenetic tree was constructed by the neighbor joining method using MEGA 7.0 software.

### Screening of Polyphenol-Producing Endophytic Fungi

Endophytic fungi were inoculated into fresh PDB and were allowed to grow for 7 days (160 rpm/min and 28°C). The culture was filtered through four layers of cheesecloth to obtain culture filtrate. Polyphenol-producing endophytic fungi were preliminarily screened by the color reaction. In brief, fermentation broth was mixed with chromogenic reagent (0.1% FeCl_3_:0.1% K_3_[Fe(CN)_6_] = 1:1) in a tube. If the color turns blue, it indicates that the fermentation broth contains polyphenols.

### Preparation of Extracts of Fermentation Broth of Endophytic Fungi

Expanded fermentation of polyphenol-producing endophytic fungi was carried out in the same culture conditions as in Section “Screening of Polyphenol-Producing Endophytic Fungi.” The culture filtrate was extracted three times with ethyl acetate, *n*-butanol, petroleum ether, and chloroform (2 × 200 mL) in a separatory funnel, respectively. The crude extracts were concentrated using a lyophilize under a vacuum at –40°C and dissolved in dimethyl sulfoxide (DMSO) to evaluate their biological activities.

### Determination of Total Phenolic Content

Total Phenolic Content (TPC) of the fungal extracts was determined using Folin–Ciocalteu (FC) assay as described by Minussi et al. (2003). In brief, after mixing deionized water (1 mL) and extract solution (1 mL) in a test tube, FC reagent (0.5 mL) was added to the mixture and allowed to react for 4 min. There was 20% Na_2_CO_3_ (1 mL) added thereafter and the mixture was diluted to 10 mL with distilled water. The color was developed in 2 h, and the absorbance was determined at 760 nm (A_760_) using a multiskan sky (Thermos Scientific, Singapore). TPC were expressed in mg equivalent in gallic acid and obtained from the regression equation: *y* = 0.0064x + 0.063 with *R*^2^ = 0.9979. All analyses were performed in triplicate.

### Antioxidant Activity

The above-mentioned fungi extracts were adjusted to six concentrations (0.2, 0.4, 0.6, 0.8, 1.0, and 3 mg/mL) for four antioxidant activity assays [2,2-diphenyl-1-picrylhydrazyl (DPPH) radical scavenging, 2,20-azino-bis radical (ABTS+⋅) scavenging, hydroxyl radical (⋅OH) scavenging, and superoxide anion radical (⋅O_2_^–^) scavenging]. Ascorbic acid (Vc) was used as the positive control.

#### 2,2-Diphenyl-1-Picrylhydrazyl Radical Scavenging Activity Assay

The DPPH assay was carried out using the method of Nuerxiati et al. (2019). Briefly, in each well of a 96-well microplate, test samples of different concentrations (100 μL) were mixed with DPPH solution (100 μL, 0.2 mM) in the dark for 30 min at room temperature. The absorbance of each well was recorded at 517 nm (A_517_). The DPPH radical scavenging activity (scavenging rate) was calculated by


Scavengingrate(%)=[1-(A-517A)0/A]max×100%

where A_*max*_ is the absorbance of 100 μL of DPPH solution in 100 μL of anhydrous ethanol at 517 nm; A_517_ is the absorbance of 100 μL of DPPH solution mixed with 100 μL of gradient sample solution; A_0_ is the absorbance of 100 μL of gradient sample solution in 100 μL of anhydrous ethanol.

#### Hydroxyl Radical Scavenging Activity Assay

The hydroxyl radical scavenging activity of the crude extracts was measured according to Smirnoff and Cumbes (1989) with some modifications. Different concentrations of the tested sample (50 μL) were added into an enzyme label plate containing a reaction solution of 50 μL of 6.0 mM FeSO_4_, 50 μL of 6.0 mM salicylic acid-ethanol, and 50 μL of 0.1% H_2_O_2_. The reaction solution was cultured in a 37°C thermostat for 30 min. The absorbance of the mixture was read at 510 nm (A_510_). The hydroxyl radical scavenging rate was calculated using the following formula:


Scavengingrate(%)=[1-(A-510A)0/A]m⁢a⁢x×100%

where A_510_ is the absorbance of a reaction solution containing the sample solution and H_2_O_2_ at 510 nm; A_*max*_ is the absorbance of a blank solution with anhydrous ethanol instead of the sample solution; A_0_ is the absorbance of a solution with distilled water instead of H_2_O_2_.

#### Superoxide Anion Radical Scavenging Assay

The scavenging activity of superoxide anion radical was estimated by pyrogallol autoxidation as described by Wang et al. (2009) with some modifications. Briefly, 0.05 mol/L Tris-HCl buffer (pH 8.2, 1.5 mL) and test sample with different concentration (0.5 mL) were mixed and incubated in a water bath at 25°C for 25 min. Afterward, 25 mM pyrogallol (200 μL) at 25°C was added. After 4 min of reaction, 8 mol/L HCl (0.25 mL) was added to terminate the reaction. The absorbance of the sample was measured at 325 nm (A_325_). The scavenging activity of superoxide anion radical scavenging rate was measured using the following equation:


Scavengingrate(%)=[1-(A-325A)0/A]m⁢a⁢x×100%

where A_325_ is the absorbance of a reaction solution containing the sample solution and pyrogallol at 325 nm; A_*max*_ is the absorbance of a blank solution with distilled water; A_0_ is the absorbance of a solution with distilled water instead of pyrogallol.

#### ABTS Radical Scavenging Activity Assay

ABTS assay of all samples was assessed as the method described by Zhao et al. (2005). The ABTS solution was formed by mixing 7 mM ABTS (0.5 mL) with 140 mM K_2_S_2_O_8_ (88 μL), and the mixture was kept at room temperature for 12 h. The ABTS radical solution was diluted with distilled water and absorbance was adjusted to 0.70 ± 0.02 at 734 nm. Then, different concentrations of extracts (150 μL) were mixed with 50 μL of ABTS solution in each well of a 96-well microplate for 6 min. The absorbance of the reaction mixture was read at 734 nm (A_734_). The scavenging activity of ABTS radical was calculated as:


Scavengingrate(%)=[1-(A-734A)0/A]m⁢a⁢x×100%

where A_*max*_ is the absorbance of ABTS solution mixed with anhydrous ethanol at 734 nm; A_734_ is the absorbance of ABTS solution mixed with the sample; A_0_ is the absorbance of the sample mixed with anhydrous ethanol.

### Antibacterial Activity

#### Determination of the Minimum Inhibitory Concentration

To evaluate the Minimum Inhibitory Concentration (MIC) (Molla et al., 2016), two gram-negative bacteria (*Escherichia coli:* ATCC25922 and *Pseudomonas aeruginosa:* ATCC9027) and two gram-positive bacteria (*Bacillus subtilis*: ATCC6633 and *Staphylococcus aureus*: ATCC6538) were used. Initially, the bacterial strains were inoculated on a sterile nutrient agar plate and incubated at 37°C for 24 h. Subsequently, the fungal extracts (0.2–4 mg/mL) were diluted in nutrient broth using the serial micro-dilution method. Bacterium (10 μL) was inoculated in EP tubes with different concentration samples. There was 0.5% DMSO used as a negative control and chloramphenicol (50 μg/L) as a positive control. Then, all EP tubes were incubated at 37°C for 24 h. To determine the MIC, 0.1 mg/ml MTT (10 μL) was used. Any color change observed from mauve to purple was considered negative for bacterial growth. The lowest concentration of endophytic fungi extracts with no color change occurred was recorded as the MIC value. All the experiments were performed in triplicate for each bacterium.

#### Determination of Minimum Bactericidal Concentration

To evaluate the Minimum Bactericidal Concentration (MBC) (da Rocha et al., 2020), 10 μL positive wells of bacteria were inoculated on nutrient agar plates at 37°C for 24 h. Then the colonies on the plate were counted, and the result with growth less than 10 colonies was considered to have a bactericidal effect.

#### Fluorescence Microscopy

Biofilm was visualized using fluorescence microscopy according to Jardak et al. (2017). *E. coli*, *P. aeruginosa*, and *S. aureus* cells were treated with fungal extracts at a concentration of 2MIC. Biofilms were grown on glass slides at 37°C for 48 h. Then, the glass slides were gently washed with physiological saline 2–3 times in order to remove non-adherent bacteria. Then, acridine orange (0.1%, w/v, dissolved in PBS 1X) was used to stain biofilms formed in glass slides. Biofilms were observed under a BX53 fluorescent microscope (Olympus, Tokyo, Japan).

### Detection of Bioactive Compounds by Liquid Chromatography-Mass Spectrometry Analysis

Extracts of endophytic fungi were analyzed by UPLC-HRMS (Waters, UPLC; Thermo, Q Exactive). The separation of compounds was achieved on an ACQUITY UPLC HSS T3 column (2.1 × 100 mm i.d) with bead size of 1.8 μm. The mobile phase was 0.05% formic acid in water (A)/acetonitrile (B) with a flow rate of 0.3 mL min^–1^. The following gradient elution program was used: 0–1 min, 5% B; 5–95% B, 2–13:50 min; 95–5% B, 13:50–16 min. For mass spectrometry (MS) detection, ionization was performed in ESI+ mode (Heater Temp 300°C; Sheath Gas Flow rate, 45arb; Aux Gas Flow Rate, 15arb; Sweep Gas Flow Rate, 1arb; spray voltage, 3.0KV; Capillary Temp, 350°C; S-Lens RF Level, 30%.) and ESI- mode (Heater Temp 300°C, Sheath Gas Flow rate,45arb; Aux Gas Flow Rate, 15arb; Sweep Gas Flow Rate, 1arb; spray voltage, 3.2KV; Capillary Temp, 350°C; S-Lens RF Level, 60%.). Full Scan (MS_1_) was performed from 70∼1050 m/z with a resolution of 70,000 and data dependent two-stage mass spectrometry (DD-MS_2_, TOPN = 10) with a resolution of 17,500. The obtained mass spectra were analyzed with Compound Discoverer 2.0 (Thermo Scientific) and the integrated mzcloud, metlin, and hmdb database were used to detect secondary metabolites in an untargeted method.

### Data Analysis

All experimental data are expressed as the mean ± SD from three independent observations. The data were analyzed by one-way analysis of variance (ANOVA) followed by Duncan’s multiple range test using the SPSS24.0 software. *P* < 0.05 was used to define statistically significant difference between the control group and the experimental group.

## Results

### Isolation and Identification of Endophytic Fungi

**FIGURE 1:**
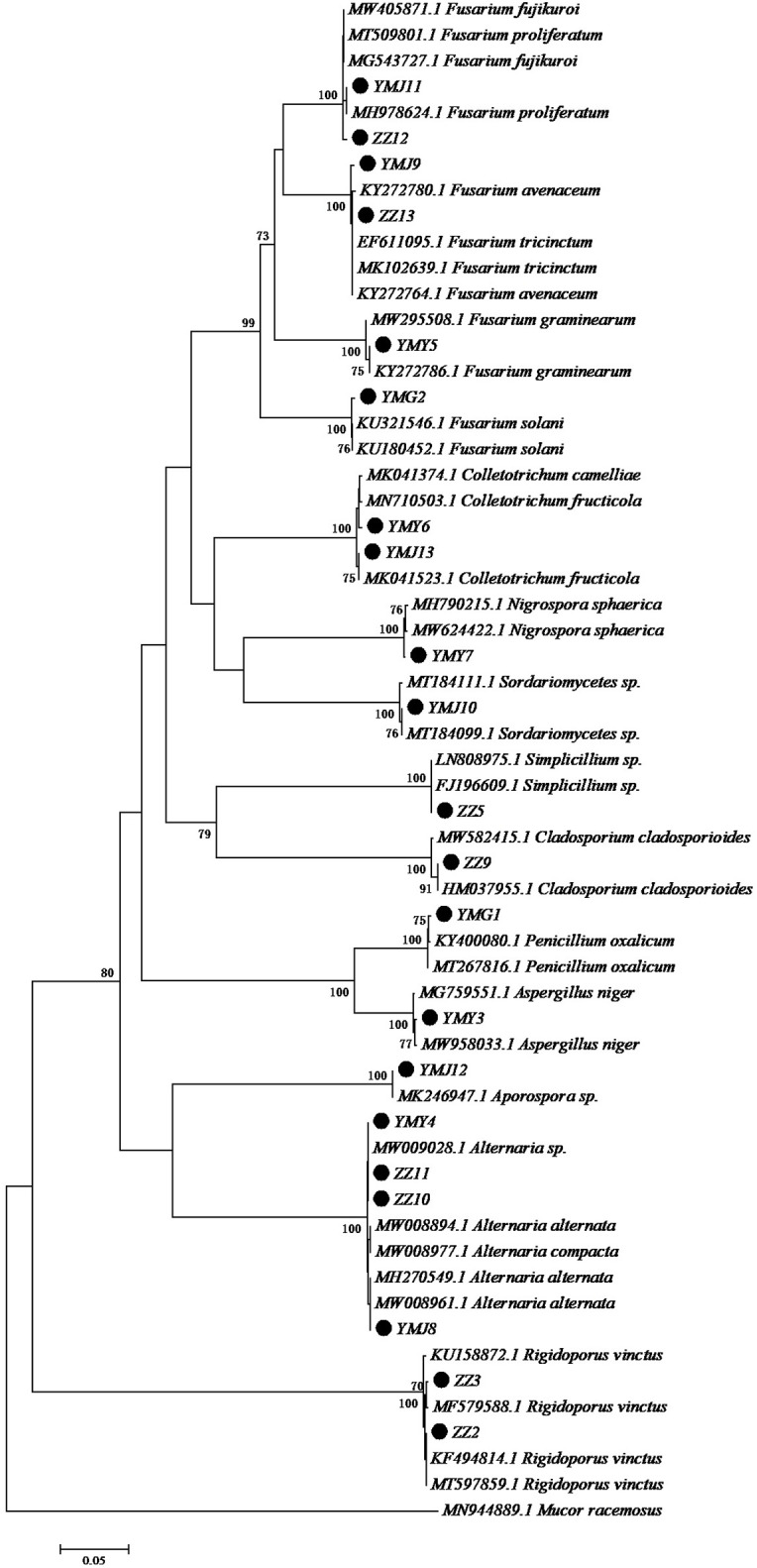
Neighbor-joining tree of the ITS sequences of the endophytic fungi associated with CX. Numbers at nodes are bootstrap scores obtained from 1000 replications. *Mucor racemosus* was used as an out group.

A total of 21 strains of endophytic fungi were isolated from different parts of CX for the first time. There were 8, 2, 5, and 6 isolates obtained from lingzi, root, leaf, and stem of CX, respectively (Table 1). All these strains were preliminarily identified by their colony morphology and microscopic examination (Supplementary Figure 1 and Supplementary Table 1). Further, their molecular identification was carried out by ITS-based rDNA sequence analysis and the closest identified matching strains were listed in Table 2. All nucleotide sequences of these isolates were no less than 99% similarity to the closest matches in the nucleotide database, except for strain ZZ2, which showed only 90.14% identity with the sequence KM268690.1 retrieved from the NCBI GenBank database (Table 2). The most common genera were *Fusarium* sp. and *Alternaria* sp. (seven isolates and four isolates, respectively) (Table 2). The phylogenetic tree of the identified endophytic fungi from CX is shown in Figure 1. The 21 isolates were assigned into 11 different genus (Figure 1), including *Rigidoporus* sp., *Aspergillus* sp., *Simplilium* sp., *Cladosporium* sp., *Alternaria* sp., *Fusarium* sp., *Penicillium* sp., *Colletotrichum* sp., *Nigrospora* sp., *Sordariomycetes* sp., and *Aporospora* sp., showing the rich biodiversity of endophytic fungi from CX. Finally, ZZ2 was characterized as *Rigidoporus* sp. at the genus level based on the observation of colony morphology and phylogenetic analysis (Supplementary Figure 1, Supplementary Table 1, and Figure 1). In addition, since Colletotrichum is a very complex genus and need for identification of it at the species level by further genomics analyses (Gupta et al., 2019), strains YMY6 and YMJ13 also were preliminarily ascribed to *Colletotrichum* sp. at the genus level.

**TABLE 1:** The result of separation of endophytic fungi from different parts of CX.

**Stage**	**Parts**	**Strain no**	**Total**
Lingzi		ZZ2, ZZ3, ZZ5, ZZ9, ZZ10, ZZ11, ZZ12, ZZ13	8
Seedling	Root	YMG1, YMG2	2
	Leaf	YMY3, YMY4, YMY5, YMY6, YMY7	5
	Stem	YMJ8, YMJ9, YMJ10, YMJ11, YMJ12, YMJ13	6

**TABLE 2:** The homologous strain of endophyte fungi from CX.

**NO**	**Genus**	**Most closely related strain**	**Ident (%)**	**Accession.**
ZZ2	*Rigidoporus* sp.	*Rigidoporus vinctus*	90.14	KF494814.1
ZZ3	*Rigidoporus* sp.	*Rigidoporus vinctus*	99.51	KM277965.1
ZZ5	*Simplicillium* sp.	*Simplicillium* sp.	100	LN808975.1
ZZ9	*Cladosporium* sp.	*Cladosporium cladosporioides*	99.42	HM037955.1
ZZ10	*Alternaria* sp.	*Alternaria alternata*	99.81	MH270549.1
ZZ11	*Alternaria* sp.	*Alternaria alternata*	99.81	MW008961.1
ZZ12	*Fusarium* sp.	*Fusarium fujikuroi*	99.62	MG543727.1
ZZ13	*Fusarium* sp.	*Fusarium tricinctum*	99.62	EF611095.1
YMG1	*Penicillium* sp.	*Penicillium oxalicum*	99.46	MT267816.1
YMG2	*Fusarium* sp.	*Fusarium solani*	99.62	KU321546.1
YMY3	*Alternaria* sp.	*Alternaria* sp.	99.63	MW009028.1
YMY4	*Alternaria* sp.	*Alternaria compacta*	99.81	MW008977.1
YMY5	*Fusarium* sp.	*Fusarium asiaticum*	99.8	KY272822.1
YMY6	*Colletotrichum* sp.	*Colletotrichum camelliae*	99.63	MK041374.1
YMY7	*Nigrospora* sp.	*Nigrospora sphaerica*	99.61	MH790215.1
YMJ8	*Alternaria* sp.	*Alternaria* sp.	99.63	MW009028.1
YMJ9	*Fusarium* sp.	*Fusarium avenaceum*	99.43	KY272780.1
YMJ10	*Sordariomycetes* sp.	*Sordariomycetes* sp.	99.08	MT184111.1
YMJ11	*Fusarium* sp.	*Fusarium proliferatum*	99.05	MH978624.1
YMJ12	*Aporospora* sp.	*Aporospora* sp.	100	MK246947.1
YMJ13	*Colletotrichum* sp.	*Colletotrichum fructicola*	99.08	MK041492.1

### Screening of Polyphenol-Producing Endophytic Fungi

Polyphenol-producing strains were screened by color reaction, as shown in Supplementary Figure 2. The fermentation broth containing polyphenols reacted a blue reaction in FeCl_3_- K_3_[Fe(CN)_6_] solution. The color reaction of *Penicillium oxalicum* (YMG1), *Simplilium* sp. (ZZ5), and *Colletotrichum* sp. (YMJ13) were blue, indicating that these strains had the potential to produce polyphenols, and were further selected for chemical and pharmacological studies.

### Determination of Total Phenolic Content

Extraction of *Penicillium oxalicum* (*P*. *oxalicum*), *Simplilium* sp., and *Colletotrichum* sp. using different solvents allowed the collection of mixture with different total phenolic contents (Table 3). The TPC of the fungal extracts ranged between 12.29 ± 3.24 and 94.58 ± 2.70 mg GAE/g with *P. oxalicum* possessing higher polyphenol content than *Simplilium* sp. and *Colletotrichum* sp. For *P. oxalicum* and *Simplicillium* sp., ethyl acetate was the most effective solvent for polyphenol extraction, and the recovered polyphenols are 94.58 ± 2.70 and 58.64 ± 3.61 mg GAE/g, respectively. As for *Colletotrichum* sp., *n*-butanol was the effective solvent for the extraction of polyphenols, with 58.96 ± 4.10 GAE/g polyphenols. However, the petroleum ether extract of *P*. *oxalicum*, *Simplicillium* sp., and *Colletotrichum* sp. contained considerably less polyphenols with 21.67 ± 5.91, 12.29 ± 3.24, and 26.14 ± 0.32 mg GAE/g, respectively. Therefore, for *Colletotrichum* sp., polar solvents allowed the recovery of more polyphenol. For *P*. *oxalicum* and *Simplicillium* sp., the medium polarity solvents were more suitable for polyphenol extraction.

**TABLE 3:** Assessment of TPC and antioxidant activity of *P*. *oxalicum*, *Simplicillium* sp., and *Colletotrichum* sp. extracts.

**Extracts**	**TPC (mg GAE/g of extract)**	**IC_50_ (mg/mL)**			
		**ABTS radical**	**Hydroxyl radical**	**DPPH radical**	**Superoxide anion radical**
***P. oxalicum***					
Vc	–	–	0.29 ± 0.03_b_	–	0.07 ± 0.01_b_
Ethyl acetate	94.58 ± 2.701_a_	0.03 ± 0.003_c_	0.38 ± 0.04_b_	0.16 ± 0.002_c_	1.41 ± 0.22_a_
*n*-Butanol	66.46 ± 8.12_b_	0.21 ± 0.04_b_	1.22 ± 0.60_a_	0.50 ± 0.05_b_	nd
Petroleum ether	21.67 ± 5.91_c_	nd	1.29 ± 1.05_a_	nd	nd
Chloroform	52.40 ± 2.71_d_	0.35 ± 0.01_a_	1.9 ± 0.94_a_	1.15 ± 0.15_a_	nd
***Simplicillium* sp.**					
Vc	–	–	0.29 ± 0.03_b_	–	0.07 ± 0.01_b_
Ethyl acetate	58.64 ± 3.61_a_	0.18 ± 0.02_c_	3.37 ± 0.38_a_	0.04 ± 0.003_d_	2.00 ± 0.11_a_
*n*-Butanol	45.62 ± 8.27_b_	0.24 ± 0.01_b_	nd	0.07 ± 0.02_c_	nd
Petroleum ether	12.29 ± 3.24_c_	1.03 ± 0.07_a_	nd	0.45 ± 0.02_a_	nd
Chloroform	16.98 ± 4.77_c_	0.43 ± 0.01_b_	nd	0.28 ± 0.01_b_	nd
***Colletotrichum* sp.**					
Vc	–	–	0.29 ± 0.03_b_	–	0.07 ± 0.01_b_
Ethyl acetate	36.75 ± 4.83_b_	0.59 ± 0.10_a_	nd	0.97 ± 0.09_b_	nd
*n*-Butanol	58.96 ± 4.10_a_	0.29 ± 0.011_c_	0.67 ± 0.05_a_	0.47 ± 0.004_c_	2.48 ± 0.52_a_
Petroleum ether	26.14 ± 0.32_c_	0.43 ± 0.02_ab_	nd	1.65 ± 0.43_a_	nd
Chloroform	25.04 ± 5.56_c_	0.47 ± 0.04_b_	nd	1.54 ± 0.1_a_	nd

*a–d: significant differences between different groups (p < 0.05).*

*nd, not detected (the result higher 4 mg/mL).*

### Antioxidant Activity

To further explore whether the extracts possess antioxidant activity, four different methods, namely ABTS radical scavenging assay, hydroxyl radical scavenging assay, DPPH radical scavenging assay, and superoxide anion radical scavenging assay, were used to evaluate the antioxidant activities of the ethyl acetate fractions, *n*-butanol fractions, petroleum ether fractions, and chloroform fractions extracted from the *P*. *oxalicum*, *Simplicillium* sp., and *Colletotrichum* sp. The different extracts of the fermentation broth of the three strains showed antioxidant activity to different degrees, and the antioxidant activities seemed to be correlated with their TPC. In addition, the ethyl acetate extract of *P*. *oxalicum* and *Simplicillium* sp., and the n-butanol extract of *Colletotrichum* sp. had the highest antioxidant activity.

**FIGURE 2:**
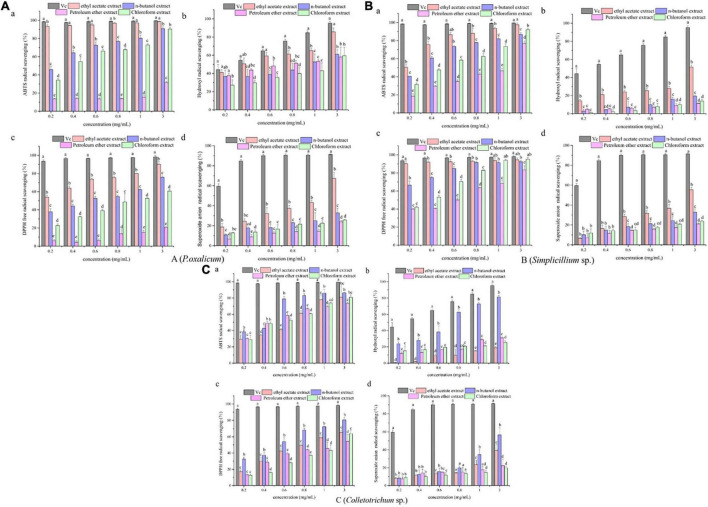
Antioxidant activities of the *P. oxalicum* **(A)**, *Simplicillium* sp. **(B)**, and *Colletotrichum* sp. **(C)** extracts. a: ABTS scavenging activity. b: Hydroxyl radical scavenging activity. c: DPPH radical scavenging activity. d: Superoxide anion radical scavenging activity. a–d: Significant differences between different groups (*p* < 0.05).

The results of antioxidant activity are shown in Figure 2 and Table 3. As seen in Figure 2A, all samples exhibited scavenging activity of ABTS, hydroxyl radical DPPH, and superoxide anion free radical at all concentrations in a concentration-dependent pattern. At 0.6 mg/mL, the scavenging rate of ABTS and hydroxyl radical of the *P*. *oxalicum* ethyl acetate extracts was similar to Vc and significantly higher than other extracts (Figures 2Aa,b; *P* < 0.05). As shown in Table 3, the ethyl acetate extracts of *P*. *oxalicum* exhibited the strongest scavenging activity (IC_50 *ABTS*__+__⋅_ = 0.03 ± 0.003 mg/mL; IC_50 ⋅__*OH*_ = 3.38 ± 0.04 mg/mL; IC_50 *DPPH*_ = 0.16 ± 0.002 mg/mL; IC_50_ ⋅o_2_^–^ = 1.41 ± 0.22 mg/mL) followed by the *n*-butanol extracts (IC_50 *ABTS*__+__⋅_ = 0.21 ± 0.04 mg/mL; IC_50 ⋅__*OH*_ = 1.22 ± 0.60 mg/mL; IC_50 *DPPH*_ = 0.50 ± 0.05 mg/mL) (*p* < 0.05). Regarding the *Simplicillium* sp. extract, the IC_50_ of the ethyl acetate extracts in the four tests were significantly lower (*P* < 0.05) compared with the other three extracts, with IC_50_ of 0.18 ± 0.002, 3.37 ± 0.38, 0.04 ± 0.003, and 2.00 ± 0.11 for ABTS radical, hydroxyl radical, DPPH radical, and superoxide anion radical, respectively (Table 3). Moreover, the DPPH radical scavenging rate of the ethyl acetate extracts of *Simplicillium* sp. was close to Vc at the same concentration, especially at 0.2, 1, and 3 mg/mL (Figure 2Bc; *p* < 0.05). The results suggest that ethyl acetate extracts of *P. oxalicum* and *Simplicillium* sp. have the strongest antioxidant activity. However, at 3 mg/mL, only the *n*-butanol extracts from *Colletotrichum* sp. showed higher 50% scavenging rate of ABTS, hydroxyl radical, DPPH, and superoxide anion radical, and the best antioxidant activity was obtained with *n*-butanol, which IC_50_ was 0.29 ± 0.011, 0.67 ± 0.05, 0.67 ± 0.05, and 2.48 ± 0.52 mg/mL, respectively (Figure 2C and Table 3). The differences observed in the radical scavenging effects among the extracts could be attributed to the differences in their polyphenol content.

### Antibacterial Activity

Antimicrobial activities of the extracts of *P*. *oxalicum*, *Simplicillium* sp., and *Colletotrichum* sp. extracts were tested against gram-positive and gram-negative bacteria (Tables 4, 5).

**TABLE 4:** Minimum inhibitory concentration (MIC) (mg/mL) of the *P*. *oxalicum*, *Simplicillium* sp., and *Colletotrichum* sp. extracts.

**Extracts**	**Gram-positive bacteria**		**Gram-negative bacteria**	
	***S. aureus***	***B. subtilis***	***E. coli***	***P. aeruginosa***
***P. oxalicum***				
Ethyl acetate	0.5	2	0.5	1
*n*-Butanol	1	nd	0.5	2
Petroleum ether	2	nd	1	nd
Chloroform	2	nd	0.5	nd
***Simplicillium sp.***				
Ethyl acetate	1	1	0.5	2
*n*-Butanol	Nd	1	1	nd
Petroleum ether	Nd	1	2	nd
Chloroform	Nd	nd	nd	nd
***Colletotrichum* sp.**				
Ethyl acetate	0.5	2	1	1
*n*-Butanol	1	2	0.5	0.5
Petroleum ether	2	nd	1	2
Chloroform	2	nd	2	2

*nd, not detected (result higher 3.00 mg/mL).*

**TABLE 5:** Minimum bactericidal concentration (MBC) (mg/mL) of the *P*. *oxalicum*, *Simplicillium* sp., and *Colletotrichum* sp. extracts.

**Extracts**	**Gram-positive bacteria**		**Gram-negative bacteria**	
	***S. aureus***	***B. subtilis***	***E. coli***	***P. aeruginosa***
***P. oxalicum***				
Ethyl acetate	2	nd	2	2
*n*-butanol	nd	nd	2	nd
Petroleum ether	nd	nd	nd	nd
Chloroform	nd	nd	nd	nd
***Simplicillium sp.***				
Ethyl acetate	2	nd	2	nd
*n*-Butanol	nd	nd	2	nd
Petroleum ether	nd	nd	nd	nd
Chloroform	nd	nd	nd	nd
***Colletotrichum sp.***				
Ethyl acetate	2	nd	1	1
*n*-Butanol	2	nd	1	2
Petroleum ether	nd	nd	nd	nd
Chloroform	nd	nd	nd	nd

*nd, not detected (result higher 3.00 mg/mL).*

The MIC of the samples was determined using the broth micro-dilution method. *P*. *oxalicum*, *Simplicillium* sp., and *Colletotrichum* sp. showed antibacterial effects against all the tested bacteria (Table 4). However, different extracts of the three fungi showed varying degrees of antibacterial and bactericidal activity depending on the solvent used (Table 4). As shown in Table 4, the ethyl acetate extracts of *P*. *oxalicum* had antibacterial effects against all the tested bacteria with a MIC between 0.5 and 2 mg/mL. However, the *n*-butanol, petroleum ether, and chloroform extracts of *P. oxalicum* had antibacterial activity only against *E. coli* and *S. aureus*, but not against antibacterial effect of *B*. *subtilis* and *P*. *aeruginosa*. Similarly, only the ethyl acetate extracts of *Simplicillium* sp. showed antibacterial effect against *E. coli, B. subtilis*, *P. aeruginosa*, and *S. aureus* with MIC of 0.5, 1, 2, and 1 mg/mL, respectively (Table 4). Regarding *Colletotrichum* sp., *n*-butanol and ethyl acetate extracts also inhibited all tested bacteria with MICs between 0.5 and 2 mg/mL (Table 4). The above results indicated that *P. oxalicum*, *Simplicillium* sp., and *Colletotrichum* sp. have a certain degree of antibacterial ability and that ethyl acetate can effectively extract bioactive compounds of *P. oxalicum*, *Simplicillium* sp., and *Colletotrichum* sp.

The MBCs of the fungal extracts are summarized in Table 5. Against *S. aureus*, *E*. *coli*, and *P. aeruginosa*, ethyl acetate extracts of the three strains had bactericidal activity, with MBCs between 1.0 and 2.0 mg/mL. In contrast, none of the extracts exhibited bactericidal activity against *B. subtilis*.

**FIGURE 3:**
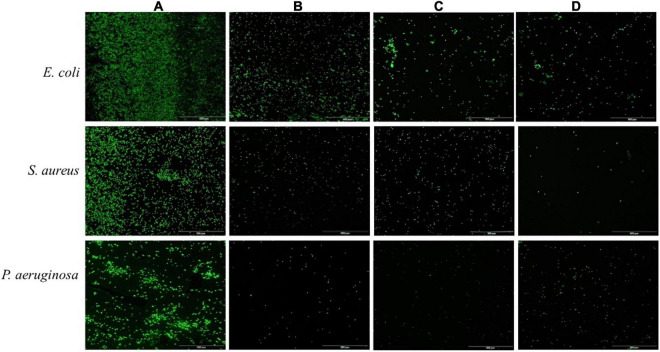
Effects of ethyl acetate extracts from endophytic fungi on cell membrane integrity of *E. coli*, *S. aureus*, and *P. aeruginosa* by fluorescence microscope. **(A)** Untreated bacterial cells; **(B)** bacterial cells treated with *P. oxalicum* extracts at 2MIC; **(C)** bacterial cells treated with *Simplicillium* sp. extracts at 2MIC; **(D)** bacterial cells treated with *Colletotrichum* sp. extracts at 2MIC. The scale bar was 100 μm.

Acridine orange can freely penetrate the cell membrane of bacterial cells, bind to nucleic acids, and emit green fluorescence. As shown in Figure 3, untreated bacterial cell maintained intact cell membrane and emitted intense green fluorescence (Figure 3A). In contrast, the green fluorescence significantly decreased after treatment with ethyl acetate extracts at a concentration of 2MIC, which indicated that the cell membranes of pathogenic bacteria (*S. aureus*, *E*. *coli*, and *P*. *aeruginosa*) induced by ethyl acetate extracts were damaged (Figures 3B–D). The above results suggested that ethyl acetate extracts can potently inhibit cell growth and differentiation by disrupting cell membranes.

### Liquid Chromatography-Mass Spectrometry

The ethyl acetate extracts of *P*. *oxalicum* and *Simplicillium* sp. and the *n*-butanol extracts of *Colletotrichum* sp. exhibited effective anti-microbial and antioxidant activity with high polyphenol content and were selected for compound analysis by liquid chromatography-mass spectrometry (LC-MS) analytical technique. Table 6 shows the retention time, molecular weight, molecular formula, M/Z, and content of the identified chemical constituents presented in the extracts. The chromatograms are shown in Supplementary Figure 3. The identified compounds included phenolic acids (caffeic acid, chlorogenic acid, ferulic acid, etc.); flavonoids (zpigenin, phloretin, luteolin, hesperetin, etc.); fatty acids (palmitic acid, oleic acid, linoleic acid); organic acid (succinic acid, citric acid, etc.); and monosaccharide [sucrose, d-(–)-fructose, and d-(+)-glucose] (Table 6). As shown in Table 6, a total of 36 compounds were identified in *P. oxalicum*, of which hesperetin was the major compound with a concentration of 36.06 μmol/g. A total of 28 compounds were identified in *Colletotrichum* sp., and its major compound was succinic acid (8.11 μmol/g). This was followed by *Simplicillium* sp. extracts with 34 identified compounds. 4-hydroxyphenylpyruvic acid (17.23 μmol/g) and d (+)-phenylactic acid (4.01 μmol/g) were major compounds in *Simplicillium* sp. extracts. All in all, phenolic and organic acids were the most abundant compounds in the extracts of the three endophytic fungi, which may be closely related to the biological activity of the secondary metabolites of endophytic fungi.

**TABLE 6:** The identification of the chemical composition of endophytic fungal extracts by LC-MS analysis.

**S/N**	**Name of identified compound**	**Adducts**	**Molecular formula**	**RT (min)**		**Endophytic fungi (μmol/g)**		
					**M/S**	***P. oxalicum***	***Colletotrichum* sp.**	***Simplicillium* sp.**
1	4-Methylcatechol	[M-H]^–^	C_7_H_8_O_2_	2.671	123.044	1.35	0.41	0.10
2	Gentisic acid	[M-H]^–^	C_7_H_6_O_4_	3.084	153.018	8.10	0.33	1.15
3	3,4-Dihydroxyphenylacetic acid	[M-H]^–^	C_8_H_8_O_4_	2.673	167.034	5.73	1.73	0.39
4	Caffeic acid	[M-H]^–^	C_9_H_8_O_4_	4.018	179.034	3.01	0.52	0.12
5	Alternariol	[M-H]^–^	C_14_H_10_O_5_	5.978	257.045	12.64	0.05	0.57
6	Isorhapontigenin	[M-H]^–^	C_15_H_14_O_4_	8.46	257.082	0.39	nd	nd
7	Apigenin	[M-H]^–^	C_15_H_10_O_5_	9.207	269.045	4.56	nd	0.06
8	Genistein	[M-H]^–^	C_15_H_10_O_5_	6.784	269.045	0.35	nd	0.01
9	Phloretin	[M-H]^–^	C_15_H_14_O_5_	4.751	273.076	1.86	nd	0.02
10	Luteolin	[M-H]^–^	C_15_H_10_O_6_	6.875	285.040	0.56	nd	0.01
11	Eriodictyol	[M-H]^–^	C_15_H_12_O_6_	7.423	287.056	0.57	nd	nd
12	Hesperetin	[M-H]^–^	C_15_H_10_O_7_	6.789	301.071	36.06	0.06	0.87
13	Neochlorogenic acid	[M-H]^–^	C_16_H_18_O_9_	3.167	353.087	1.14	0.14	0.34
14	Cryptochlorogenic acid	[M-H]^–^	C_16_H_18_O_9_	3.671	353.087	0.93	0.21	1.21
15	Chlorogenic acid	[M-H]^–^	C_16_H_18_O_9_	3.94	353.087	0.11	0.04	0.12
16	Pyruvic acid	[M-H]^–^	C_3_H_4_O_3_	0.917	87.007	1.06	2.25	0.69
17	Succinic acid	[M-H]^–^	C_4_H_6_O_4_	1.412	117.018	16.22	8.11	3.47
18	Benzoic acid	[M-H]^–^	C_7_H_6_O_2_	4.41	121.028	1.19	0.92	0.46
19	Itaconic acid	[M-H]^–^	C_5_H_6_O_4_	1.192	129.018	1.19	0.50	0.36
20	2,4-Dihydroxybenzoic acid	[M-H]^–^	C_7_H_6_O_4_	4.05	153.018	0.83	0.19	0.03
21	D(+)-Phenyllactic acid	[M-H]^–^	C_9_H_10_O_3_	4.775	165.055	1.98	2.27	4.01
22	Theanine	[M-H]^–^	C_7_H_14_N_2_O_3_	3.898	173.092	1.10	nd	0.29
23	Indoleacetic acid	[M-H]^–^	C_10_H_9_NO_2_	5.582	175.023	0.22	0.12	0.01
24	2-Isopropylmalic acid	[M-H]^–^	C_7_H_12_O_5_	3.623	175.060	5.95	0.85	1.29
25	Esculetin	[M-H]^–^	C_9_H_6_O_4_	5.524	177.018	0.01	0.02	2.94
26	D-(-)-Fructose	[M-H]^–^	C_6_H_12_O_6_	0.874	179.055	1.53	0.36	1.91
27	D-(+)-Glucose	[M-H]^–^	C_6_H_12_O_6_	1.099	179.055	1.67	0.45	2.84
28	Citric acid	[M-H]^–^	C_6_H_8_O_7_	0.918	191.019	18.49	5.56	1.97
29	Ferulic acid	[M-H]^–^	C_10_H_10_O_4_	4.757	193.050	1.17	0.11	1.10
30	Palmitic acid	[M-H]^–^	C_16_H_32_O_2_	13.345	255.232	0.93	0.13	0.23
31	Alpha-linolenic acid	[M-H]^–^	C_18_H_30_O_2_	12.177	277.217	1.57	0.03	0.08
32	Linoleic acid	[M-H]^–^	C_18_H_32_O_2_	12.795	279.232	4.70	0.28	0.70
33	Oleic acid	[M-H]^–^	C_18_H_34_O_2_	13.512	253.217	0.99	0.20	0.33
34	Maleic acid	[M-H]^–^	C_4_H_4_O_4_	1.329	115.002	7.81	0.14	0.13
35	Juzirine	[M-H]^–^	C_8_H_11_N_9_O_3_	3.112	280.093	4.69	nd	0.02
36	4-Hydroxyphenylpyruvic acid	[M-H]^–^	C_9_H_8_O_4_	4.364	179.034	0.25	1.20	17.23

*nd, not detect.*

## Discussion

The CX is a well-known Chinese medicinal plant with diverse phytochemicals such as ferulic acid, ligustrazine, and ligustilide, which contribute to its medicinal properties (Yuan et al., 2020a). Endophytic fungi are not only one of the most important elements in plant micro-ecosystems but also a storehouse for natural products, including antibiotics, antioxidants, and anticancer agents, which could be an alternative source of secondary metabolites for medical and pharmacological applications (Strobel et al., 2002). A previous research showed that the different ages and tissues of host plants may influence the species composition of endophytic fungus community (Sieber, 2007). In this study, a total of 21 endophytic fungi belonging to 11 different genera were isolated from different parts of CX. This is similar to what was observed in CX by Wang et al. (2008), who also found *Penicillium*, *Fusarium*, *Aspergillus*, *Alternaria*, etc. Some species, such as *Nigrospora sphaerica* and *Sordariomycetes* sp., are exclusive to different plant parts. The tissue specificity of a particular endophyte suggests that certain species perpetuate within the specific chemistry or texture of a particular tissue due to the differences in anatomical structure and physiological conditions (Park et al., 2012). Only *Fusarium* sp. was present in the four sites studied. The pleiotropic colonization of *Fusarium* sp. may be related to its secreted metabolites (Pappas et al., 2018), which can protect host plants from biotic or abiotic stresses. The *Aporospora* sp. and *Sordariomycetes* sp. isolated from CX in this study have not been reported previously (Hu et al., 2016). These results increase the diversity of endophytic fungi in CX.

Polyphenols have hydroxyl groups and play a vital role in anti-oxidation, scavenging free radicals and other pharmacological activities (Gangwar et al., 2014). Therefore, the discovery of polyphenol-producing endophytic fungi could help meet the needs of the pharmaceutical and food industry. For instance, Govindappa et al. (2013) reported that organic extracts of *Penicillium* sp. present a large amount of phenolic compounds that act as antioxidants and could be used for medicinal or food preservation. Fill et al. (2010) discovered an effective antioxidant compound, namely, phenylpropanoid amide, isolated from the endophytic fungus *Penicillium brasilianum*. Similarly, Tian et al. (2020) found that *Simplicillium* sp. ethyl acetate extracts have excellent antioxidant activity, which contains various chemical components. In the present study, *P. oxalicum*, *Simplicillium* sp., and *Colletotrichum* sp. isolated from CX were polyphenol-producing strains, which deserve further study.

Different solvents can recover compounds of different contents and components in the current study, we found polar and medium polarity solvents are more favorable to the extraction of low and high molecular weight polyphenols in endophytic fungus fermentation broth compared to those of non-polar solvent. This finding is consistent with a previous study (Scholz and Rimpler, 1989). The highest antioxidant activity was observed in the *P*. *oxalicum* extracts, which may be linked to the concentrations of the chemical compounds and the compositions identified in the fungal extracts. Hesperidin was the main phenolic compound in the extracts of *P*. *oxalicum*, which has powerful radical scavenging activity, and it augmented the antioxidant cellular defenses *via* the ERK/Nrf2 signaling pathway as well (Parhiz et al., 2015). However, compared to the potency of the standard antioxidants Vc, the extracts of the endophytic fungi showed lower antioxidant activity. Moreover, the antioxidants used as standards are purified molecules, while the endophytic fungi extracts represent a group of mixtures containing different concentrations of substances.

Endophytic fungi have also been recognized as producers of antimicrobial substances, such as Simplicildone A and Botryorhodine C isolated from the fungus *Simpilcillium* sp. PSU-H41 displayed antibacterial against *S. aureus* (Saetang et al., 2017). In our study, we observed that three isolated endophytic fungi showed bacteriostatic activity against the test strain, whereas different extract parts of endophytic fungi showed varying degrees of antibacterial effect. Data from the literature demonstrating that the antibacterial effects of endophytic fungi extracts are related to the extraction solvent (Mefteh et al., 2018), which corroborates our results. Among the endophytic fungi studied, *P*. *oxalicum* stood out for the best antibacterial and bactericidal activity, which could be attributed to organic acids and phenolic compounds. Among the compounds identified in the extracts of *P*. *oxalicum*, citric acid and hesperidin were the major compounds, followed by alternariol, succinic acid, and gentisic acid, which were higher than those of *Simplicillium* sp. and *Colletotrichum* sp. Gu (2009) have reported that alternariol from endophytic fungus *Alternaria brassicicola* possesses antibacterial properties. The antibacterial activity of citric acid against bacteria was attributed to its physical and chemical properties, reduced production of extracellular aggregates, and hydrophobicity of the cell surface (Zhang et al., 2019).

Antibacterial compounds can act on bacterial cell walls, plasma membranes, protein synthesis, nucleic acid metabolism, and DNA, thereby killing pathogenic bacteria (Li et al., 2015). Among the numerous antibacterial compounds, we have emphasized phenolic compounds, which exhibit great structural diversity, such as the presence, number and substitution position of hydroxyl groups, and the length of saturated side chains, which confer antibacterial activity to these compounds (Cushnie and Lamb, 2011; Rempe et al., 2017). For example, Rasch (2002) found that phenolic acid inhibited the activity of ribonucleic acid reductase, a key enzyme required for DNA synthesis, and that bacterial DNA synthesis was blocked. Our findings provided further evidence that extracts of *P. oxalicum*, *Simplicillium* sp., and *Colletotrichum* sp. displayed anti-biofilm activity against the tested strains with an almost complete destruction of the biofilm matrix.

The *P. oxalicum*, *Simplicillium* sp., and *Colletotrichum* sp. isolated from CX are promising endophytic fungi, and the chemical composition of endophytic fungus extracts have been identified by LC-MS. Some of the identified chemical components have been reported to have significant antimicrobial, antioxidant, and other biological activities, while others have not been reported, such as juzirine. In addition, some of these compounds with biological activity were isolated and identified from plants rather than microorganisms. For example, hesperidin, as an anti-inflammatory and antioxidant drug (Li and Schluesener, 2017), is traditionally extracted from abundant natural citrus fruits using large amounts of ethanol or methanol solvents, which is not conducive to sustainable development in the pharmaceutical industry. In our study, *P. oxalicum* can produce abundant secondary metabolites including phenolic acids, flavonoids, and fatty acids. Phenolic acids and flavonoids are important secondary metabolites that exert therapeutic effects (Winter et al., 2017; Adamczak et al., 2019). Fatty acids are able to reduce and resist the oxidative stress of free radicals through a series of physiological and biochemical reactions (Gülçin, 2012; Mocking et al., 2018). Previous studies have reported the isolation and characterization of Penicilisorin (Arunpanichlert et al., 2010) and *P*-hydroxybenzaldehyde (Schmeda-Hirschmann et al., 2005), an antibacterial compound from the endophytic fungi *Penicillium sclerotiorum* PSU-A13 and *Penicillium janczewskii*, respectively. Similarly, a coumarone antioxidant, pestacin and isopestacin, were isolated from the endophytic fungus *Pestalotiopsis microspora* (Strobel et al., 2002). These results suggested that *P. oxalicum* from CX containing many active compounds makes it a promising source of molecules for future studies. Interestingly, we also found that *P*. *oxalicum* strain can produce ferulic acid, chlorogenic acid, and caffeic acid, just like their host plant, indicating that *P. oxalicum* may play an important role in affecting the quality and quantity of the CX through a specific fungus-host interaction (Ding et al., 2018). In the future, because of the fast growth rate of endophytic fungi and the advantage of easy operation during fermentation (Ludwig-Müller, 2015), these active substances can be obtained by fermentation rather than from the plant tissues itself. Moreover, the production capacity of secondary metabolites of *P*. *oxalicum* and their biological effects could be increased by optimizing cultivation conditions or genetic engineering. In conclusion, in order to use the endophytic fungus of CX in medicine, more detailed studies are needed in future research.

## Conclusion

In conclusion, the present study expanded the diversity of endophytic fungal species in CX and confirmed antioxidant and antimicrobial activities of several endophytic fungi extracts, among which the ethyl acetate extract of the endophytic fungus *P. oxalicum* stood out. The antioxidant and antibacterial activities of the *P*. *oxalicum* extracts are attributed to the phenolic compounds. Together, this study suggests that *P. oxalicum* from CX is a promising source for detection of natural antioxidants and antibacterial compounds. However, in order to use this endophytic fungus in medicine, more detailed studies need to be conducted.

## Data Availability Statement

The original contributions presented in the study are included in the article/Supplementary Material, further inquiries can be directed to the corresponding author/s.

## Author Contributions

ZT and YQ conceived and designed the experiments, performed the experiments, and prepared figures and/or tables. WC, ZZ, WL, QL, HY, YL, and YW performed the experiments, prepared the figures and/or tables. HuC conceived and designed the experiments, drafted the work, and approved the final draft. YX and YC analyzed the data and drafted the work. TB and HoC analyzed the data and revised it critically for important content. All authors contributed to the article and approved the submitted version.

## Conflict of Interest

The authors declare that the research was conducted in the absence of any commercial or financial relationships that could be construed as a potential conflict of interest.

## Publisher’s Note

All claims expressed in this article are solely those of the authors and do not necessarily represent those of their affiliated organizations, or those of the publisher, the editors and the reviewers. Any product that may be evaluated in this article, or claim that may be made by its manufacturer, is not guaranteed or endorsed by the publisher.

## References

[B1] Adamczak A. Ożarowski M. Karpiński T. M. (2019). Antibacterial activity of some flavonoids and organic acids widely distributed in plants. *J. Clin. Med.* 9:109. 10.3390/jcm9010109 31906141 PMC7019947

[B2] Aly A. H. Debbab A. Proksch P. (2011). Fungal endophytes: unique plant inhabitants with great promises. *Appl. Microbiol. Biotechnol.* 90 1829–1845. 10.1007/s00253-011-3270-y 21523479

[B3] Anderson C. J. Kendall M. M. (2017). *Salmonella enterica* serovar typhimurium strategies for host adaptation. *Front. Microbiol.* 8:1983. 10.3389/fmicb.2017.01983 29075247 PMC5643478

[B4] Apel K. Hirt H. (2004). Reactive oxygen species: metabolism, oxidative stress, and signal transduction. *Annu. Rev. Plant Biol.* 55 373–399. 10.1146/annurev.arplant.55.031903.141701 15377225

[B5] Arunpanichlert J. Rukachaisirikul V. Sukpondma Y. Phongpaichit S. Tewtrakul S. Rungjindamai N. (2010). Azaphilone and isocoumarin derivatives from the endophytic fungus *Penicillium sclerotiorum* PSU-A13. *Chem. Pharm. Bull.* 58 1033–1036. 10.1248/cpb.58.1033 20686255

[B6] Chen Z. Zhang C. Gao F. Fu Q. Fu C. He Y. (2018). A systematic review on the rhizome of *Ligusticum chuanxiong* Hort. (Chuanxiong). *Food Chem. Toxicol.* 119 309–325. 10.1016/j.fct.2018.02.050 29486278

[B7] Cushnie T. P. T. Lamb A. J. (2011). Recent advances in understanding the antibacterial properties of flavonoids. *Int. J. Antimicrob. Agents* 38 99–107. 10.1016/j.ijantimicag.2011.02.014 21514796

[B8] da Rocha P. D. S. Paula V. M. B. Olinto S. C. F. Dos Santos E. L. Souza K. de P. (2020). Diversity, chemical constituents and biological activities of endophytic fungi isolated from *Schinus terebinthifolius* raddi. *Microorganisms* 8 1–13. 10.3390/microorganisms8060859 32517286 PMC7356110

[B9] Del Giudice P. (2020). Skin infections caused by *Staphylococcus aureus*. *Acta Derm. Venereol.* 100 208–215. 10.2340/00015555-3466 32207539 PMC9128951

[B10] Desai N. Sharma R. Makker K. Sabanegh E. Agarwal A. (2009). Physiologic and pathologic levels of reactive oxygen species in neat semen of infertile men. *Fertil. Steril.* 92 1626–1631. 10.1016/j.fertnstert.2008.08.109 18937945

[B11] Ding C. H. Wang Q. B. Guo S. Wang Z. Y. (2018). The improvement of bioactive secondary metabolites accumulation in *Rumex gmelini* Turcz through co-culture with endophytic fungi. *Braz. J. Microbiol.* 49 362–369. 10.1016/j.bjm.2017.04.013 29254631 PMC5913822

[B12] Fill T. P. da Silva B. F. Rodrigues-Fo E. (2010). Biosynthesis of phenylpropanoid amides by an endophytic *Penicillium brasilianum* found in root bark of *Melia azedarach*. *J. Microbiol. Biotechnol.* 20 622–629. 20372037

[B13] Gangwar M. Gautam M. K. Sharma A. K. Tripathi Y. B. Goel R. K. Nath G. (2014). Antioxidant capacity and radical scavenging effect of polyphenol rich *Mallotus philippenensis* fruit extract on human erythrocytes: an in vitro study. *Sci. World J.* 2014:279451. 10.1155/2014/279451 25525615 PMC4261553

[B14] Ge H. Chen Y. Chen J. Tian J. Liang X. Chen L. (2018). Evaluation of antioxidant activities of ethanol extract from *Ligusticum* subjected to in-vitro gastrointestinal digestion. *Food Chem. Toxicol.* 119 417–424. 10.1016/j.fct.2017.12.035 29274897

[B15] Govindappa M. Channabasava R. Sunil Kumar K. R. Pushpalatha K. C. (2013). Antioxidant activity and phytochemical screening of crude endophytes extracts of *Tabebuia argentea* Bur. & K. Sch. *Am. J. Plant Sci.* 3 1641–1652. 10.4236/ajps.2013.48198

[B16] Gu W. (2009). Bioactive metabolites from alternaria brassicicola ML-P08, an endophytic fungus residing in malus halliana. *World J. Microbiol. Biotechnol.* 25 1677–1683. 10.1007/s11274-009-0062-y

[B17] Gülçin I. (2012). Antioxidant activity of food constituents: an overview. *Arch. Toxicol.* 86 345–391. 10.1007/s00204-011-0774-2 22102161

[B18] Gupta D. R. Kabir M. K. Hassan O. Sabir A. A. Mahmud N. U. Surovy M. Z. (2019). First report of anthracnose crown rot of strawberry caused by *Colletotrichum siamense* in Rajshahi District of Bangladesh. *Plant Dis.* 103:1775.

[B19] Hu R. Gong G. Ye H. Yao L. Huang X. (2016). Isolation and identification of *Ligusticum chuanxiong* rhizome Fungi of Dujiangyan in Sichuan. *Southwest China J. Agric. Sci.* 29 1238–1240. 10.16213/j

[B20] Huang W. Y. Cai Y. Z. Xing J. Corke H. Sun M. (2007). A potential antioxidant resource: endophytic fungi from medicinal plants. *Econ. Bot.* 61 14–30.

[B21] Jardak M. Elloumi-Mseddi J. Aifa S. Mnif S. (2017). Chemical composition, anti-biofilm activity and potential cytotoxic effect on cancer cells of *Rosmarinus officinalis* L. essential oil from Tunisia. *Lipids Health Dis.* 16 1–10. 10.1186/s12944-017-0580-9 28969677 PMC5625792

[B22] Jia M. Chen L. Xin H. L. Zheng C. J. Rahman K. Han T. (2016). A friendly relationship between endophytic fungi and medicinal plants: a systematic review. *Front. Microbiol.* 7:906. 10.3389/fmicb.2016.00906 27375610 PMC4899461

[B23] Kahl R. Kappus H. (1993). Toxikologie der synthetischen antioxidantien BHA und BHT im vergleich mit dem natürlichen antioxidans Vitamin E. *Z. Lebensmittel Untersuchung Forschung* 196 329–338. 10.1007/BF01197931 8493816

[B24] Kimmig A. Hagel S. Weis S. Bahrs C. Löffler B. Pletz M. W. (2021). Management of *Staphylococcus aureus* bloodstream infections. *Front. Med.* 7:616524. 10.3389/fmed.2020.616524 33748151 PMC7973019

[B25] Li C. Schluesener H. (2017). Health-promoting effects of the citrus flavanone hesperidin. *Crit. Rev. Food Sci. Nutr.* 57 613–631. 10.1080/10408398.2014.906382 25675136

[B26] Li G. Kusari S. Kusari P. Kayser O. Spiteller M. (2015). Endophytic *Diaporthe* sp. LG23 produces a potent antibacterial tetracyclic triterpenoid. *J. Nat. Prod.* 78 2128–2132. 10.1021/acs.jnatprod.5b00170 26186257

[B27] Ludwig-Müller J. (2015). Plants and endophytes: equal partners in secondary metabolite production? *Biotechnol. Lett.* 37 1325–1334. 10.1007/s10529-015-1814-4 25792513

[B28] Marseglia L. Manti S. D’Angelo G. Nicotera A. Parisi E. Di Rosa G. (2015). Oxidative stress in obesity: a critical component in human diseases. *Int. J. Mol. Sci.* 16 378–400. 10.3390/ijms16010378 25548896 PMC4307252

[B29] Martin R. M. Bachman M. A. (2018). Colonization, infection, and the accessory genome of *Klebsiella pneumoniae*. *Front. Cell. Infect. Microbiol.* 8:4. 10.3389/fcimb.2018.00004 29404282 PMC5786545

[B30] Mefteh F. B. Daoud A. Bouket A. C. Thissera B. Kadri Y. Cherif-Silini H. (2018). Date palm trees root-derived endophytes as fungal cell factories for diverse bioactive metabolites. *Int. J. Mol. Sci.* 19:1986. 10.3390/ijms19071986 29986518 PMC6073733

[B31] Minussi R. C. Rossi M. Bologna L. Cordi L. Rotilio D. Pastore G. M. (2003). Phenolic compounds and total antioxidant potential of commercial wines. *Food Chem.* 82 409–416. 10.1016/S0308-8146(02)00590-3

[B32] Mocking R. J. T. Assies J. Ruhé H. G. Schene A. H. (2018). Focus on fatty acids in the neurometabolic pathophysiology of psychiatric disorders. *J. Inherit. Metab. Dis.* 41 597–611. 10.1007/s10545-018-0158-3 29524021

[B33] Molla Y. Nedi T. Tadesse G. Alemayehu H. Shibeshi W. (2016). Evaluation of the in vitro antibacterial activity of the solvent fractions of the leaves of *Rhamnus prinoides* L’Herit (Rhamnaceae) against pathogenic bacteria. *BMC Complement. Altern. Med.* 16:287. 10.1186/s12906-016-1279-6 27527076 PMC4986379

[B34] Nischitha R. Shivanna M. B. (2021). Antimicrobial activity and metabolite profiling of endophytic fungi in *Digitaria bicornis* (Lam) Roem. and Schult. and *Paspalidium flavidum* (Retz.) A. Camus. *3 Biotech* 11:53. 10.1007/s13205-020-02590-x 33489672 PMC7801544

[B35] Noinart J. Buttachon S. Dethoup T. Gales L. Pereira J. A. Urbatzka R. (2017). A new ergosterol analog, a new bis-anthraquinone and anti-obesity activity of anthraquinones from the marine sponge-associated fungus *Talaromyces stipitatus* KUFA 0207. *Mar. Drugs* 15:139. 10.3390/md15050139 28509846 PMC5450545

[B36] Nuerxiati R. Abuduwaili A. Mutailifu P. Wubulikasimu A. Rustamova N. Jingxue C. (2019). Optimization of ultrasonic-assisted extraction, characterization and biological activities of polysaccharides from *Orchis chusua* D. Don (Salep). *Int. J. Biol. Macromol.* 141 431–443. 10.1016/j.ijbiomac.2019.08.112 31445150

[B37] Padhi S. Masi M. Panda S. K. Luyten W. Cimmino A. Tayung K. (2020). Antimicrobial secondary metabolites of an endolichenic *Aspergillus niger* isolated from lichen thallus of *Parmotrema ravum*. *Nat. Prod. Res.* 34 2573–2580. 10.1080/14786419.2018.1544982 30600725

[B38] Pappas M. L. Liapoura M. Papantoniou D. Avramidou M. Kavroulakis N. Weinhold A. (2018). The beneficial endophytic fungus *Fusarium solani* strain K alters tomato responses against spider mites to the benefit of the plant. *Front. Plant Sci.* 9:1603. 10.3389/fpls.2018.01603 30459791 PMC6232530

[B39] Parhiz H. Roohbakhsh A. Soltani F. Rezaee R. Iranshahi M. (2015). Antioxidant and anti-inflammatory properties of the citrus flavonoids hesperidin and hesperetin: an updated review of their molecular mechanisms and experimental models. *Phytother. Res.* 29 323–331. 10.1002/ptr.5256 25394264

[B40] Park Y. H. Lee S. G. Ahn D. J. Kwon T. R. Park S. U. Lim H. S. (2012). Diversity of Fungal endophytes in various tissues of panax ginseng meyer cultivated in Korea. *J. Ginseng Res.* 36 211–217. 10.5142/jgr.2012.36.2.211 23717122 PMC3659578

[B41] Pinheiro E. A. A. Pina J. R. S. Feitosa A. O. Carvalho J. M. Borges F. C. Marinho P. S. B. (2017). Bioprospecting of antimicrobial activity of extracts of endophytic fungi from Bauhinia guianensis. *Rev. Argent. Microbiol.* 49 3–6. 10.1016/j.ram.2016.08.005 28094064

[B42] Ran X. Ma L. Peng C. Zhang H. Qin L. P. (2011). *Ligusticum chuanxiong* Hort: a review of chemistry and pharmacology. *Pharm. Biol.* 49 1180–1189. 10.3109/13880209.2011.576346 22014266

[B43] Rasch M. (2002). The influence of temperature, salt and pH on the inhibitory effect of reuterin on *Escherichia coli*. *Int. J. Food Microbiol.* 72 225–231. 10.1016/S0168-1605(01)00637-7 11845821

[B44] Rempe C. S. Burris K. P. Lenaghan S. C. Stewart C. N. (2017). The potential of systems biology to discover antibacterial mechanisms of plant phenolics. *Front. Microbiol.* 8:422. 10.3389/fmicb.2017.00422 28360902 PMC5352675

[B45] Saetang P. Rukachaisirikul V. Phongpaichit S. Preedanon S. Sakayaroj J. Borwornpinyo S. (2017). Depsidones and an α-pyrone derivative from *Simpilcillium* sp. PSU-H41, an endophytic fungus from *Hevea brasiliensis* leaf. *Phytochemistry* 143 115–123. 10.1016/j.phytochem.2017.08.002 28803995

[B46] Schmeda-Hirschmann G. Hormazabal E. Astudillo L. Rodriguez J. Theoduloz C. (2005). Secondary metabolites from endophytic fungi isolated from the Chilean gymnosperm *Prumnopitys andina* (Lleuque). *World J. Microbiol. Biotechnol.* 21 27–32. 10.1007/s11274-004-1552-6

[B47] Scholz E. Rimpler H. (1989). Proanthocyanidins from *Krameria triandra* root. *Planta Med.* 55 379–384. 10.1055/s-2006-962032 2813572

[B48] Shah S. Shrestha R. Maharjan S. Selosse M. A. Pant B. (2019). Isolation and characterization of plant growth-promoting endophytic fungi from the roots of *Dendrobium moniliforme*. *Plants* 8:5. 10.3390/plants8010005 30597827 PMC6359427

[B49] Sharma M. Sharma R. (2016). Drugs and drug intermediates from fungi: striving for greener processes. *Crit. Rev. Microbiol.* 42 322–338. 10.3109/1040841X.2014.947240 25159041

[B50] Sieber T. N. (2007). Endophytic fungi in forest trees: are they mutualists? *Fungal Biol. Rev.* 21 75–89. 10.1016/j.fbr.2007.05.004

[B51] Sim Y. Shin S. (2008). Combinatorial anti-Trichophyton effects of *Ligusticum chuanxiong* essential oil components with antibiotics. *Arch. Pharm. Res.* 31 497–502. 10.1007/s12272-001-1184-7 18449508

[B52] Smirnoff N. Cumbes Q. J. (1989). Hydroxyl radical scavenging activity of compatible solutes. *Phytochemistry* 28 1057–1060. 10.1016/0031-9422(89)80182-7

[B53] Strobel G. Ford E. Worapong J. Harper J. K. Arif A. M. Grant D. M. (2002). Isopestacin, an isobenzofuranone from *Pestalotiopsis microspora*, possessing antifungal and antioxidant activities. *Phytochemistry* 60 179–183. 10.1016/S0031-9422(02)00062-6 12009322

[B54] Tanvir R. Javeed A. Bajwa A. G. (2017). Endophyte bioprospecting in South Asian medicinal plants: an attractive resource for biopharmaceuticals. *Appl. Microbiol. Biotechnol.* 101 1831–1844. 10.1007/s00253-017-8115-x 28168318

[B55] Thomer L. Schneewind O. Missiakas D. (2016). Pathogenesis of *Staphylococcus aureus* bloodstream infections. *Annu. Rev. Pathol.* 11 343–364. 10.1146/annurev-pathol-012615-044351 26925499 PMC5068359

[B56] Tian T. T. Li Q. R. Gan S. Q. Chang C. R. Shen X. C. (2020). Protective effect of *Simplicillium* sp. Ethyl acetate extract against high glucose-induced oxidative stress in HUVECs. *Evid. Based Complement. Alternat. Med.* 2020:5172765. 10.1155/2020/5172765 32879632 PMC7448235

[B57] Valko M. Leibfritz D. Moncol J. Cronin M. T. D. Mazur M. Telser J. (2007). Free radicals and antioxidants in normal physiological functions and human disease. *Int. J. Biochem. Cell Biol.* 39 44–84. 10.1016/j.biocel.2006.07.001 16978905

[B58] Valko M. Rhodes C. J. Moncol J. Izakovic M. Mazur M. (2006). Free radicals, metals and antioxidants in oxidative stress-induced cancer. *Chem. Biol. Interact.* 160 1–40. 10.1016/j.cbi.2005.12.009 16430879

[B59] Wang A. N. Yi X. W. Yu H. F. Dong B. Qiao S. Y. (2009). Free radical scavenging activity of *Lactobacillus fermentum* in vitro and its antioxidative effect on growing-finishing pigs. *J. Appl. Microbiol.* 107 1140–1148. 10.1111/j.1365-2672.2009.04294.x 19486423

[B60] Wang Y. L. Yan Z. Y. Guo X. H. Song J. Chen X. Wan D. G. (2008). Isolation and identification of endophytic fungi from *Ligusticum*. *China J. Chin. Mater. Med.* 33 999–1001. 10.3321/j 18652341

[B61] Winter A. N. Brenner M. C. Punessen N. Snodgrass M. Byars C. Arora Y. (2017). Comparison of the neuroprotective and anti-inflammatory effects of the anthocyanin metabolites, protocatechuic acid and 4-hydroxybenzoic acid. *Oxid. Med. Cell. Longev.* 2017:6297080. 10.1155/2017/6297080 28740571 PMC5504963

[B62] Wu L. Han T. Li W. Jia M. Xue L. Rahman K. (2013). Geographic and tissue influences on endophytic fungal communities of taxus chinensis var. mairei in China. *Curr. Microbiol.* 66 40–48. 10.1007/s00284-012-0235-z 23053484

[B63] Wu Y. Z. Zhang H. W. Sun Z. H. Dai J. G. Hu Y. C. Li R. (2018). Bysspectin A, an unusual octaketide dimer and the precursor derivatives from the endophytic fungus *Byssochlamys spectabilis* IMM0002 and their biological activities. *Eur. J. Med. Chem.* 145 717–725. 10.1016/j.ejmech.2018.01.030 29353723

[B64] Yuan X. Han B. Feng Z. M. Jiang J. S. Yang Y. N. Zhang P. C. (2020a). Chemical constituents of *Ligusticum chuanxiong* and their anti-inflammation and hepatoprotective activities. *Bioorgan. Chem.* 101:104016. 10.1016/j.bioorg.2020.104016 32599365

[B65] Yuan X. Han B. Feng Z. M. Jiang J. S. Yang Y. N. Zhang P. C. (2020b). Three new compounds from the rhizome of *Ligusticum chuanxiong* and their anti-inflammation activities. *J. Asian Nat. Prod. Res.* 22 920–926. 10.1080/10286020.2020.1803291 32820957

[B66] Zhang Q. Q. Zhang Y. H. Cai F. Y. Liu X. L. Chen X. H. Jiang M. (2019). Comparative antibacterial and antibiofilm activities of garlic extracts, nisin, ε−polylysine, and citric acid on *Bacillus subtilis*. *J. Food Process. Preserv.* 43:e14179. 10.1111/jfpp.14179

[B67] Zhang X. Yan H. W. Feng Z. M. Yang Y. N. Jiang J. S. Zhang P. C. (2020). Neophathalides A and B, two pairs of unusual phthalide analog enantiomers from: *Ligusticum chuanxiong*. *Organ. Biomol. Chem.* 18 5453–5457. 10.1039/d0ob01014f 32638801

[B68] Zhao L. X. Xu L. H. Jiang C. L. (2012). *Methods for the Study of Endophytic Microorganisms from Traditional Chinese Medicine Plants*, 1st Edn. Amsterdam: Elsevier Inc., 10.1016/B978-0-12-404634-4.00001-2 23084931

[B69] Zhao X. Sun H. Hou A. Zhao Q. Wei T. Xin W. (2005). Antioxidant properties of two gallotannins isolated from the leaves of *Pistacia weinmannifolia*. *Biochim. Biophys. Acta Gen. Subj.* 1725 103–110. 10.1016/j.bbagen.2005.04.015 15925448

[B70] Zimta A. A. Cenariu D. Irimie A. Magdo L. Nabavi S. M. Atanasov A. G. (2019). The role of Nrf2 activity in cancer development and progression. *Cancers* 11 1–26. 10.3390/cancers11111755 31717324 PMC6896028

