# Supplementary material for: Diversity, Chemical Constituents, and Biological Activities of Endophytic Fungi Isolated From Ligusticum chuanxiong Hort
Source: Front Microbiol. 2021 Nov 17;12:771000. doi: 10.3389/fmicb.2021.771000 (PMC8636053; doi:10.3389/fmicb.2021.771000)
Supplement: Supplementary file 1 [file Data_Sheet_1.docx]

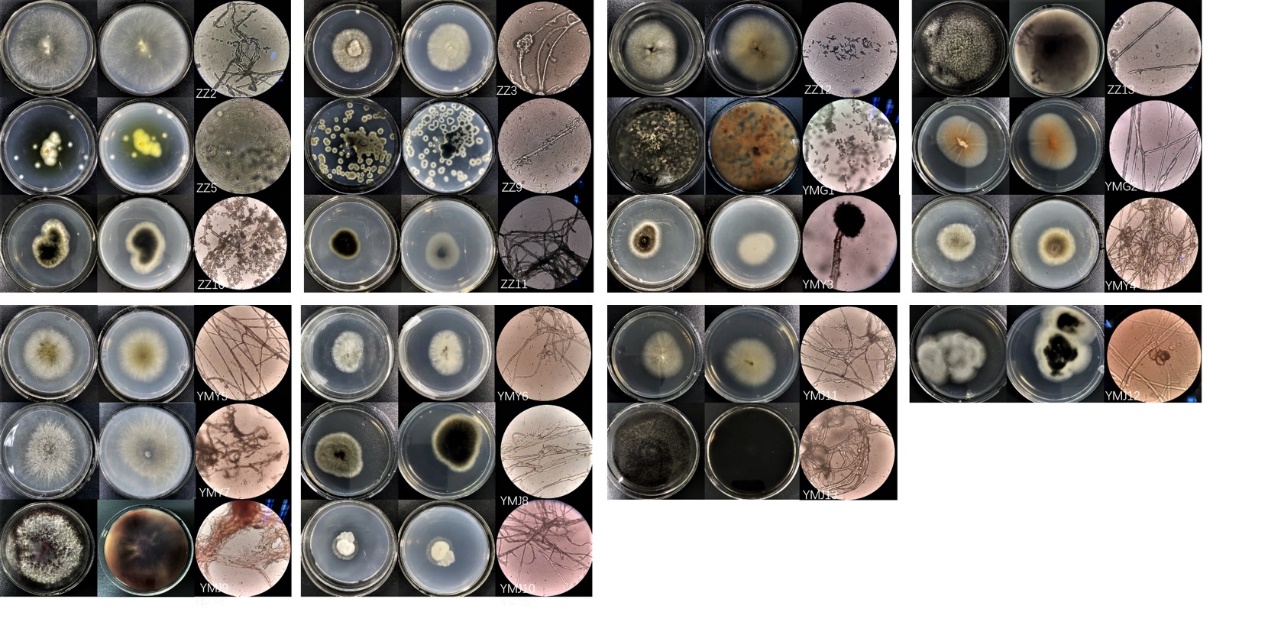


**Figure S1.** Microscopic and morphological characteristics images of endophytic fungi isolated from CX.


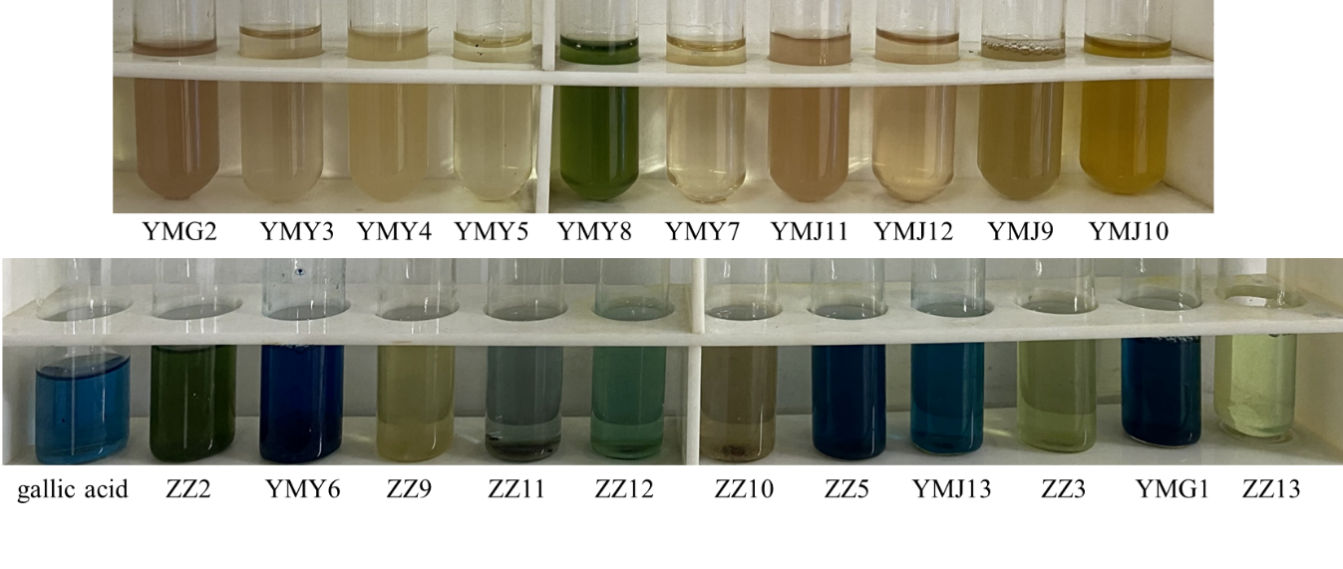


**Figure S2**. color reaction of the polyphenol-producing endophytic fungi

**
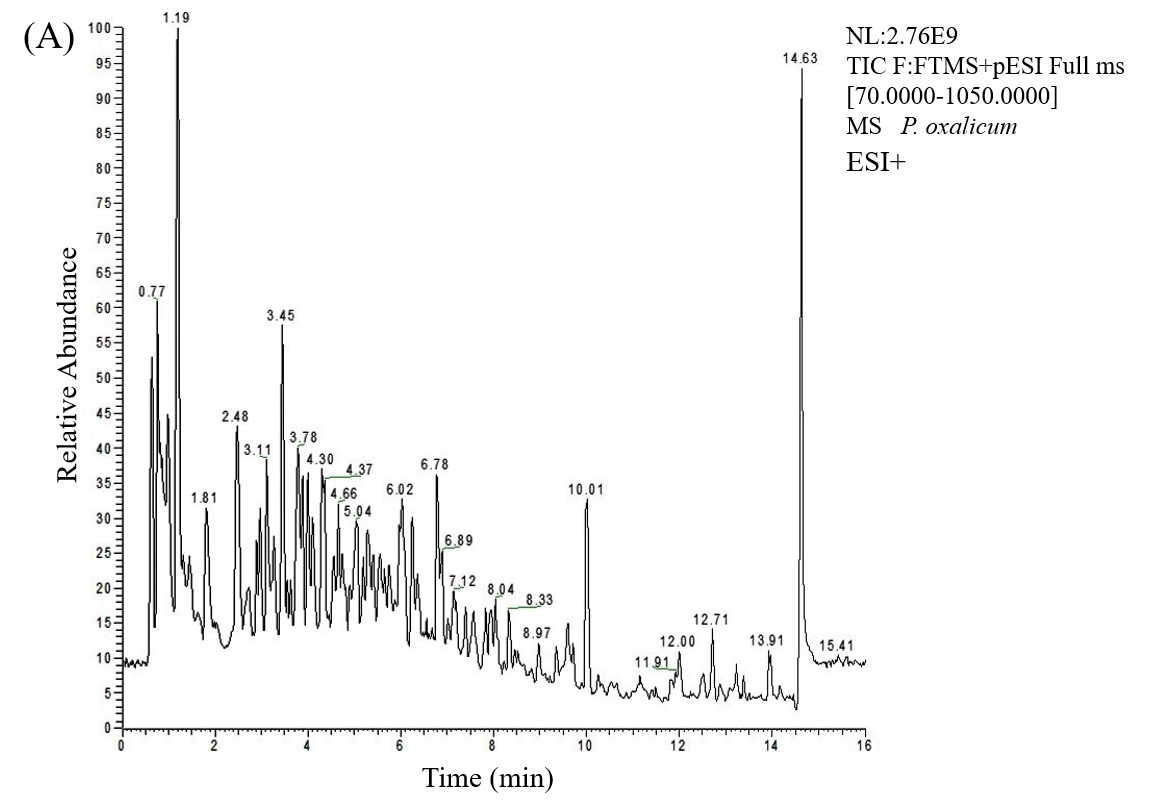

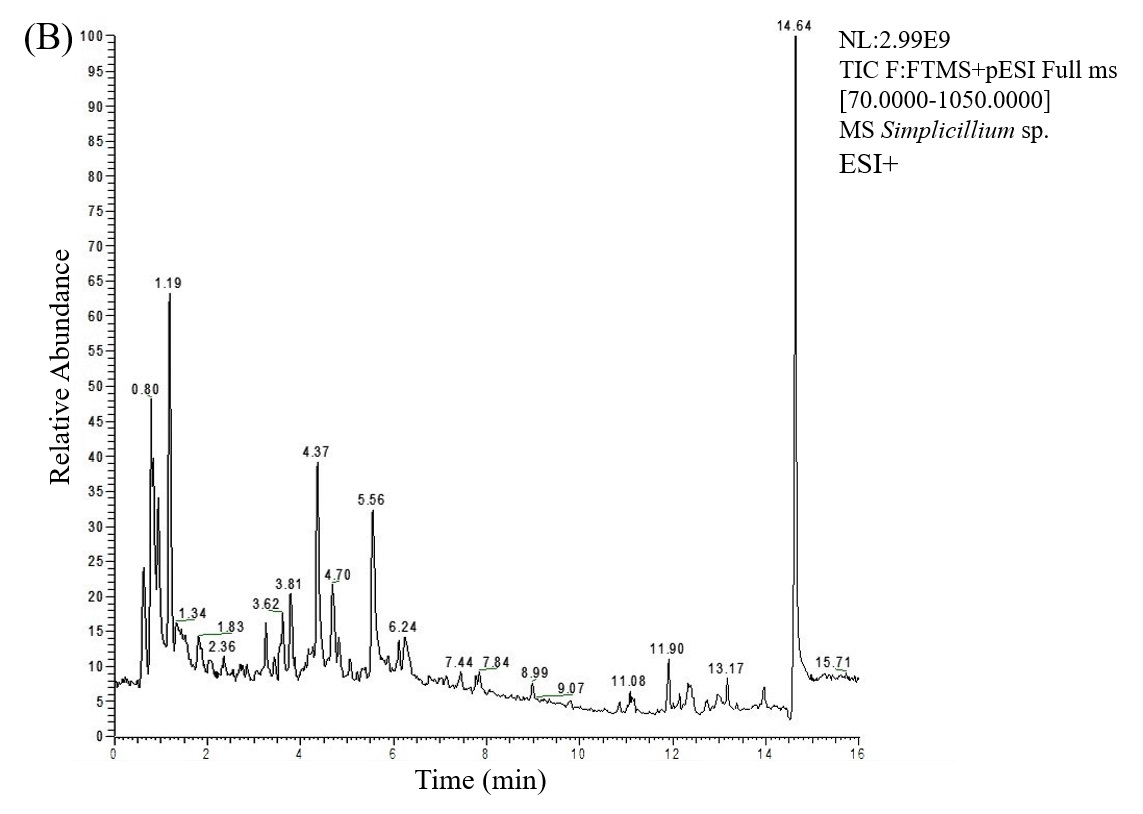

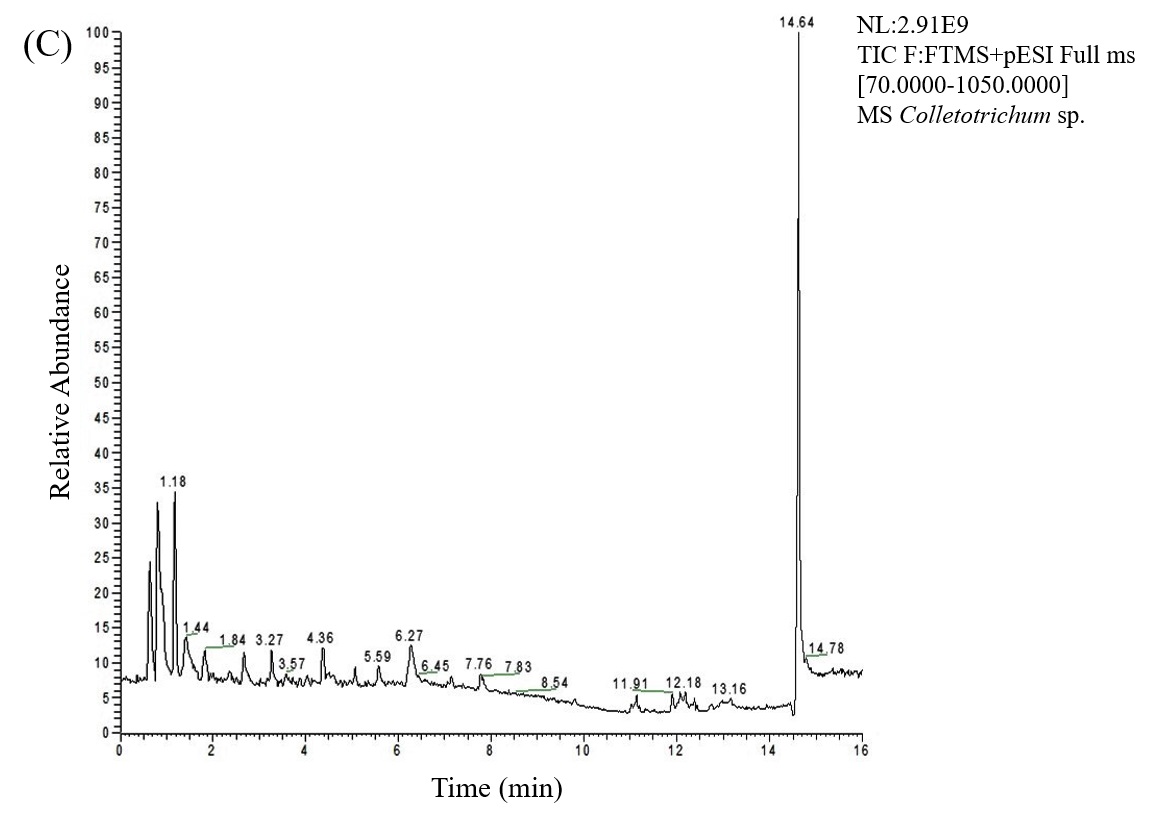
Figure S3.** LC/MS chromatogram of fungal extracts. **(A)** LC/MS chromatogram of *P. oxalicum* extracts; **(B)** LC/MS chromatogram of *Simplicillium* sp*.* extracts; **(C)** LC/MS chromatogram of *Colletotrichum* sp. extracts.

Table S1. Morphological observation of endophytic fungi isolated from CX

| Endophyte | colour | Morphology |
| --- | --- | --- |
| ZZ2 | White | filamentous |
| ZZ3 | White | highly filamentous |
| ZZ5 | Yellow and white | highly sporogenous |
| ZZ9 | Dark green | slightly filamentous |
| ZZ10 | white and green | highly sporogenous |
| ZZ11 | Green brown | slightly filamentous |
| ZZ12 | White | sporogenous |
| ZZ13 | Mauve and white | filamentous |
| YMG1 | Dark green | highly sporogenous |
| YMG2 | Orange | filamentous |
| YMY3 | Blackish | white filaments with black spores |
| YMY4 | yellowish-brown | highly filamentous |
| YMY5 | Yellow with White background | velvety and filamentous |
| YMY6 | White | highly filamentous |
| YMY7 | Dark green with brown background | filamentous |
| YMJ8 | white | filamentous |
| YMJ9 | white | filamentous |
| YMJ10 | White | velvety and filamentous |
| YMJ11 | White | filamentous |
| YMG12 | White and Dark green | Filamentous and sporogenous |
| YMG13 | White and Dark green | filamentous |
