# Supplementary figures and images for: Diversity, Chemical Constituents, and Biological Activities of Endophytic Fungi Isolated From Ligusticum chuanxiong Hort
Source: Front Microbiol. 2021 Nov 17;12:771000. doi: 10.3389/fmicb.2021.771000 (PMC8636053; doi:10.3389/fmicb.2021.771000)

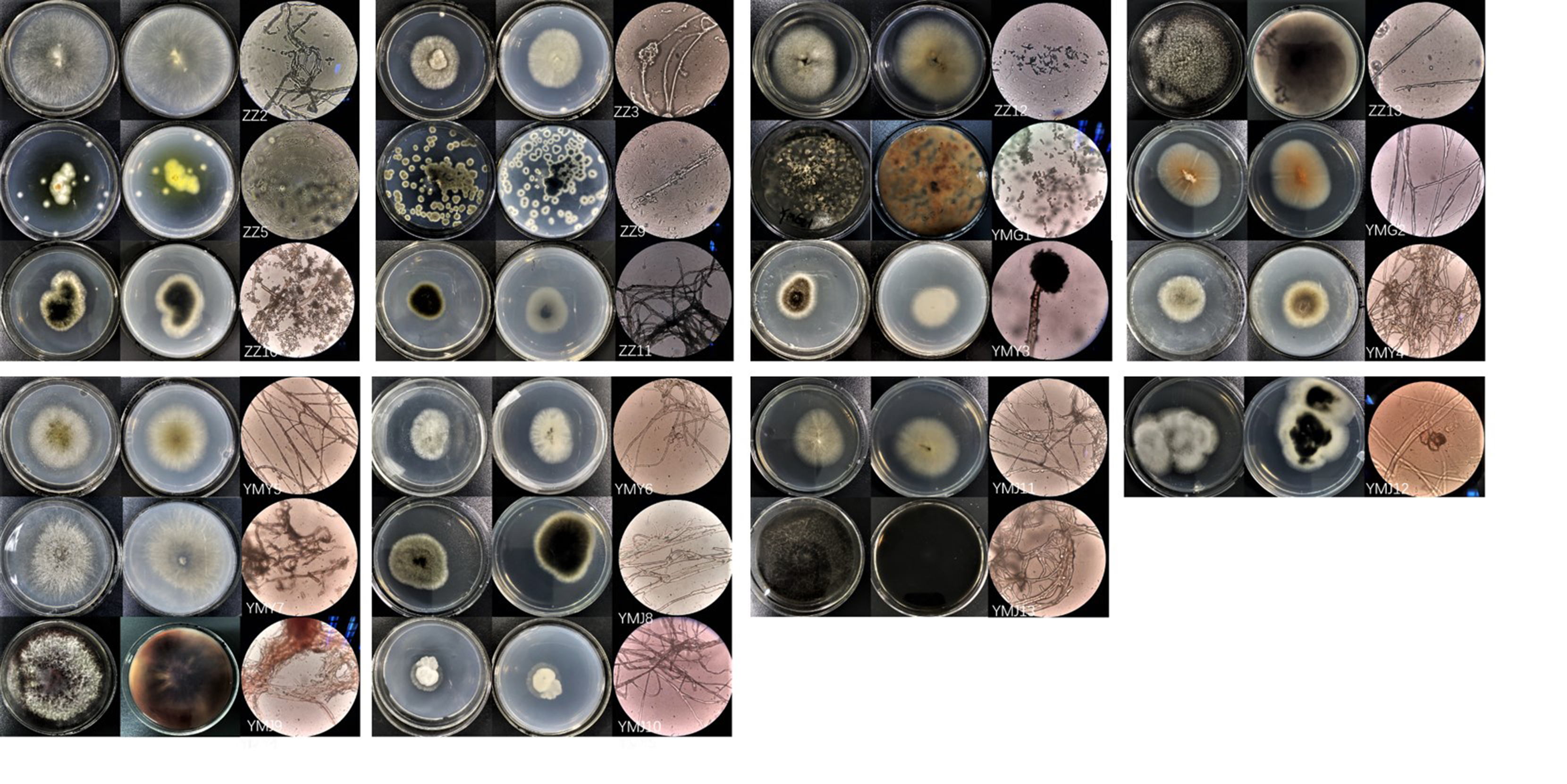

Supplement: Supplementary file 2 [file Image_1.JPEG]

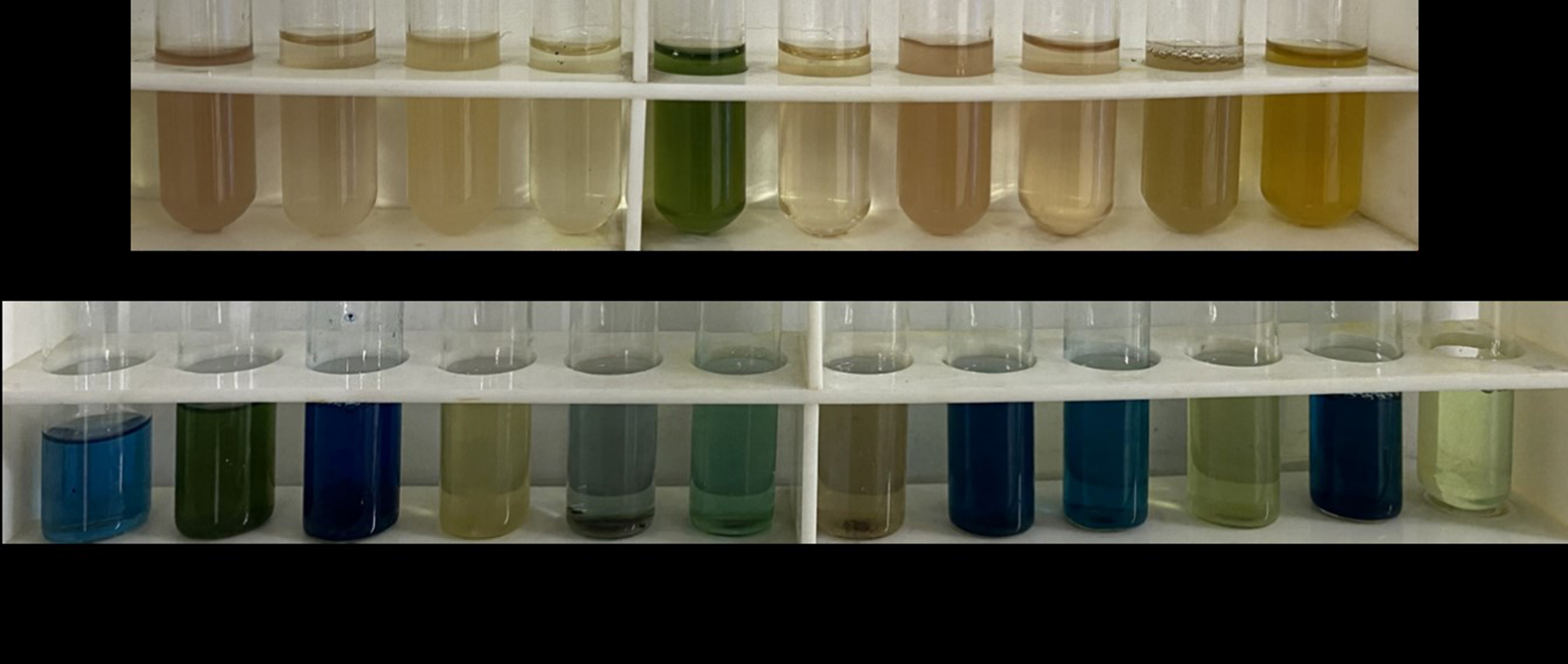

Supplement: Supplementary file 3 [file Image_2.JPEG]

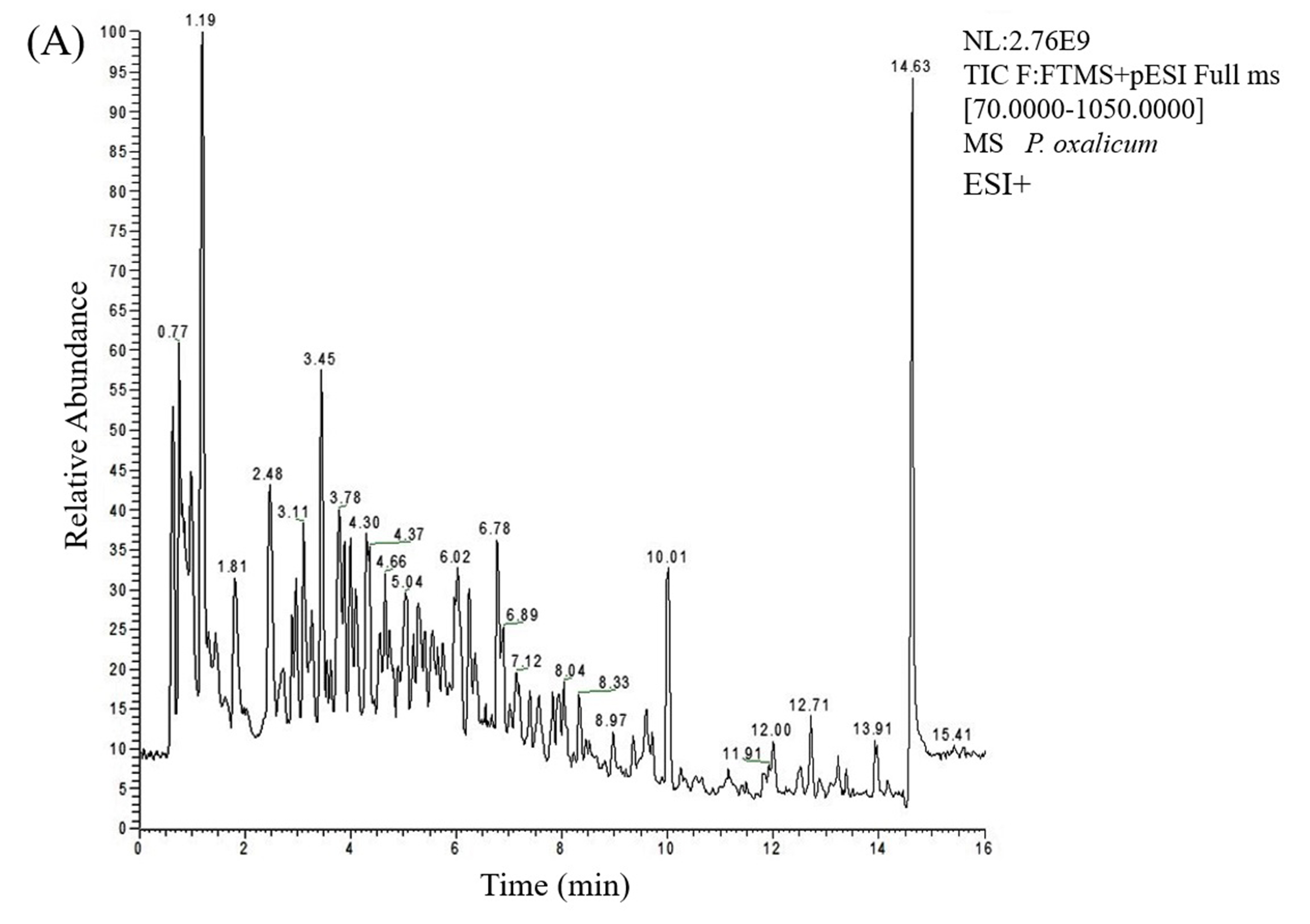

Supplement: Supplementary file 4 [file Image_3.JPEG]

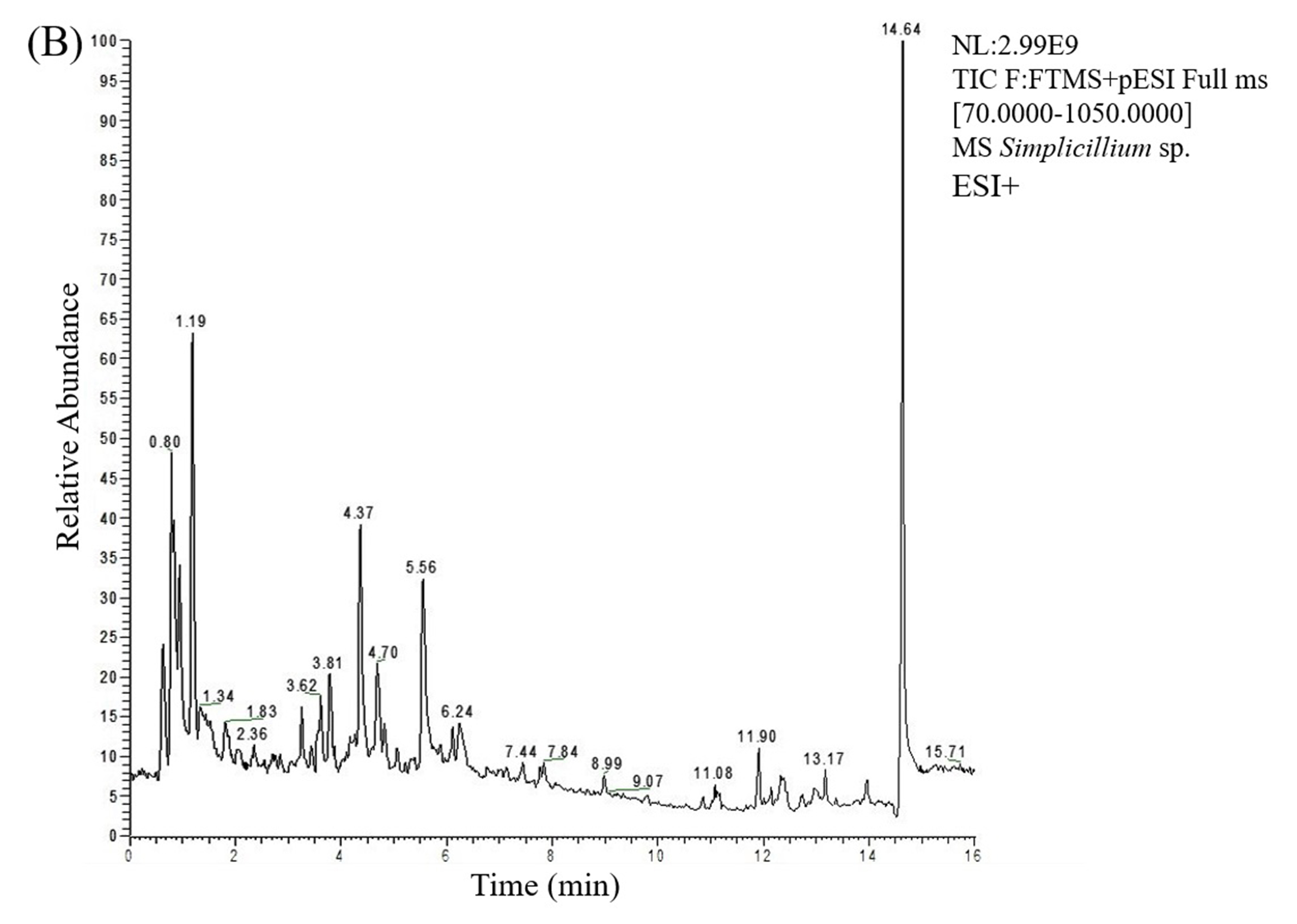

Supplement: Supplementary file 5 [file Image_4.JPEG]

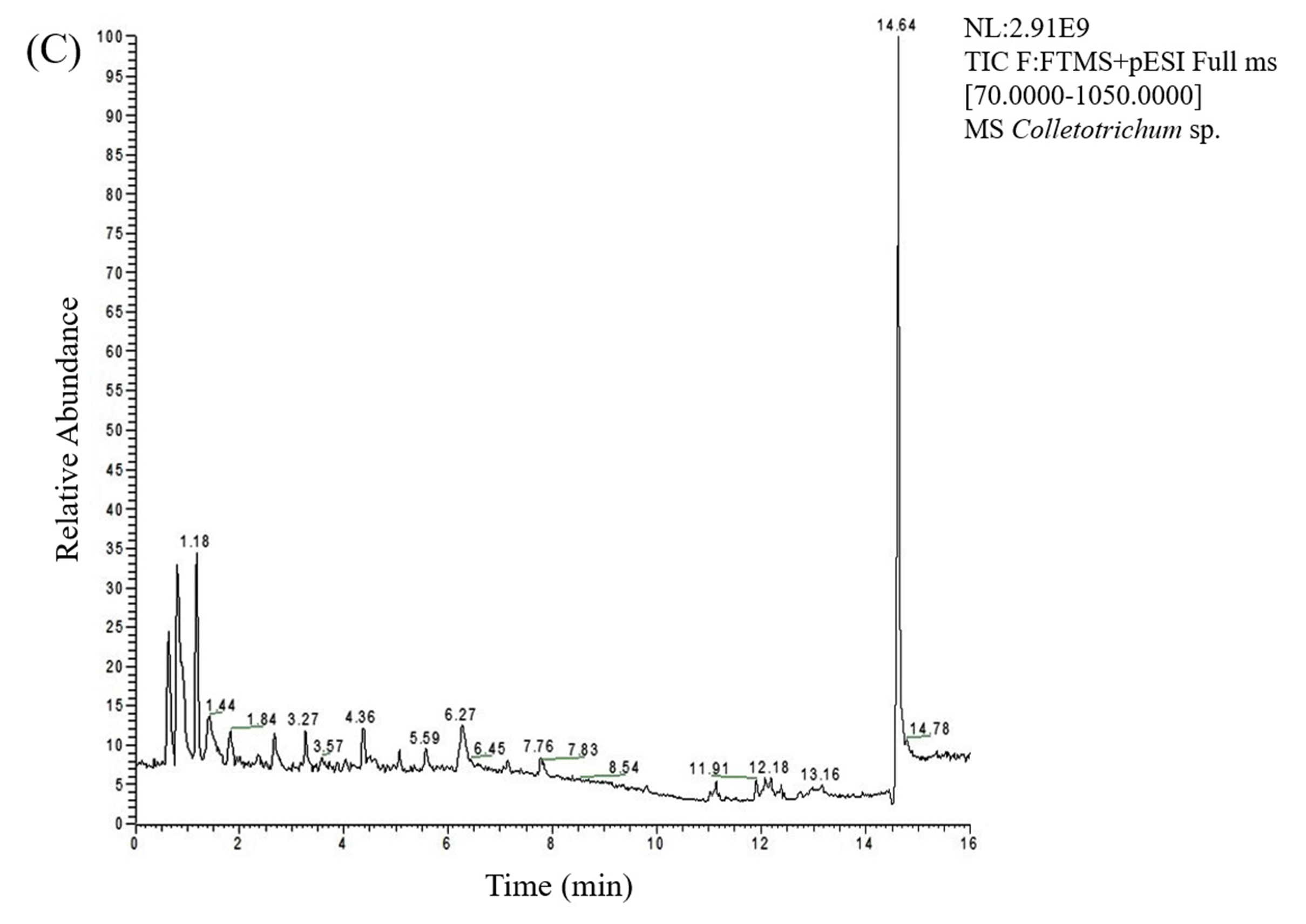

Supplement: Supplementary file 6 [file Image_5.JPEG]
